# Involvement of PARP1 in the regulation of alternative splicing

**DOI:** 10.1038/celldisc.2015.46

**Published:** 2016-02-16

**Authors:** Elena Matveeva, John Maiorano, Qingyang Zhang, Abdallah M Eteleeb, Paolo Convertini, Jing Chen, Vittoria Infantino, Stefan Stamm, Jiping Wang, Eric C Rouchka, Yvonne N Fondufe-Mittendorf

**Affiliations:** 1 Department of Molecular and Cellular Biochemistry, University of Kentucky, Lexington, KY, USA; 2 Department of Molecular Biosciences, Northwestern University, Evanston, IL, USA; 3 Department of Computer Engineering and Computer Science, University of Louisville, Louisville, KY, USA; 4 Department of Science, University of Basilicata, Potenza, Italy

**Keywords:** Epigenetics, cotranscriptional splicing, gene regulation, chromatin, PARP1

## Abstract

Specialized chromatin structures such as nucleosomes with specific histone modifications decorate exons in eukaryotic genomes, suggesting a functional connection between chromatin organization and the regulation of pre-mRNA splicing. Through profiling the functional location of Poly (ADP) ribose polymerase, we observed that it is associated with the nucleosomes at exon/intron boundaries of specific genes, suggestive of a role for this enzyme in alternative splicing. Poly (ADP) ribose polymerase has previously been implicated in the PARylation of splicing factors as well as regulation of the histone modification H3K4me3, a mark critical for co-transcriptional splicing. In light of these studies, we hypothesized that interaction of the chromatin-modifying factor, Poly (ADP) ribose polymerase with nucleosomal structures at exon–intron boundaries, might regulate pre-mRNA splicing. Using genome-wide approaches validated by gene-specific assays, we show that depletion of PARP1 or inhibition of its PARylation activity results in changes in alternative splicing of a specific subset of genes. Furthermore, we observed that PARP1 bound to RNA, splicing factors and chromatin, suggesting that Poly (ADP) ribose polymerase serves as a gene regulatory hub to facilitate co-transcriptional splicing. These studies add another function to the multi-functional protein, Poly (ADP) ribose polymerase, and provide a platform for further investigation of this protein’s function in organizing chromatin during gene regulatory processes.

## Introduction

Alternative splicing is a cellular process that serves to markedly increase the transcriptome and protein biodiversity within eukaryotic cells [1]. In humans, ~95% of multi-exonic genes are alternatively spliced [2, 3]. Alternative splicing events (ASEs) direct tissue-, cell type- and developmental stage-specific gene expression patterns in eukaryotes [4]. Alternative splicing decisions have important roles in many cellular processes, ranging from sex determination in fruit flies to programmed cell death in human cells, and are implicated in human disease [6, 5].

Alternative splicing is regulated by the binding of trans-acting factors to their target sites on pre-mRNA. These trans-acting factors promote or reduce the usage of a particular splice site. Emerging data suggest that the information present at these splice sites and the binding of these factors *in vivo* may not be sufficient to define exons or regulate alternative splicing [7]. This has led to the ‘co-transcriptional splicing hypothesis’ [8], which suggests that splicing and transcription occur at the same time, with local chromatin structure being responsible for the cross-talk between transcription and splicing. Building on this idea, several studies showed that nucleosomes and/or specific histone modifications affect both the association of splicing factors (SFs) with chromatin and the efficiency of the splicing process [8–10].

The nucleosome, the basic repeating unit of chromatin, consists of 147 bp of DNA wrapped around a histone octamer; two copies each of histone H2A, H2B, H3 and H4. The location of nucleosomes on the eukaryotic genome regulates cellular processes that require DNA to transcribe, replicate, recombine and repair DNA. Although the roles of nucleosomes positioned at promoters have been widely studied in transcriptional regulation, the roles of nucleosomes in splicing regulation are less well understood [11, 12]. The positioning of nucleosomes at exons [13, 14] is dependent on several factors including the intrinsic DNA sequence [15, 16], DNA methylation levels [17, 18] and histone modifications [19]. Indeed, nucleosomes regulate RNA polymerase elongation kinetics, thus aiding in the recognition of weak splice sites [7, 17]. These nucleosomes typically associate with DNA that has a high GC content, high DNA methylation pattern and specific histone post-translational modifications (PTMs), which are all factors that influence nucleosome stability [7, 17, 20–23]. In support of a splicing regulatory role of histone PTMs, data in yeast show elevated transcription levels are associated with reduced histone occupancy. In addition, the transcription-associated H3K36me3 modification is reduced at alternatively spliced exons compared with constitutive exons [22, 24].

As alternative splicing appears to occur co-transcriptionally *in vivo*, we hypothesize that factors affecting chromatin stability and dynamics regulate alternative splicing. Although the roles of histone PTMs and DNA methylation in co-transcriptional splicing have been extensively studied, the role of other chromatin modulators in this process is less studied. One potential modulator is poly (ADP)-ribose polymerase 1 (PARP1), which is known to remodel chromatin through PARylation (addition of poly (ADP) ribose moieties) of histones to regulate transcription. Through profiling of PARP1 chromatin-binding sites, we found sharp occupancy peaks for PARP1 at internal intron/exon boundaries, suggesting a role in pre-mRNA splicing. Our studies suggest that PARP1 acts as a structural chromatin entity and an adapter molecule bridging chromatin and RNA, and as a recruiter of SFs. These studies comprise the first comprehensive genome-wide determination of PARP1 in mediating gene regulation at the splicing level.

## Results

We assessed the genomic distribution of PARP1 in S2 *Drosophila* cells by nucleosome-chromatin immunoprecipitation using PARP1 antibody followed by deep sequencing (nuc-ChIP-seq) (Supplementary Figure S1). The *Drosophila* system provides a convenient model to test the effect of PARP1 on gene regulation as *Drosophila* contains only one PARP1 gene and a tankyrase, compared with at least 18 different PARP genes in humans [25, 26].

### PARP1 preferentially binds active promoters

**Figure 1:**
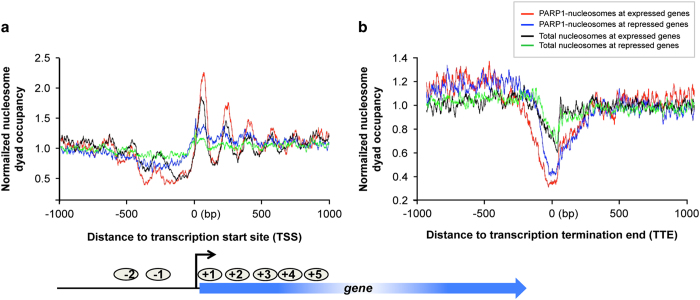
PARP1 is enriched around the promoters of active genes and depleted at the ends of genes. Dyad density plot of PARP1-bound nucleosomes and total nucleosomes in S2 cells around (**a**) transcription start sites (TSSs) and (**b**) transcription termination ends (TTEs). To control for the difference in the total number of tags, dyad density scores are normalized by the average density over the genome.

Previous studies using ChIP-chip experiments as well our recent nuc-ChIP-seq show that PARP1 binds to active promoter regions in human cells [27, 28]. We sought to determine whether this is true in the *Drosophila* genome, where the presence of a single gene permits a higher resolution nuc-ChIP-seq analysis. Using this analysis, we examined the distribution of PARP1-nucleosome reads within 2 kb upstream and downstream of annotated transcription start sites (TSSs), as described in the Materials and Methods section. We observed that PARP1 associates with the +1 and +2 nucleosomes of active promoters (Figure 1a) and not with the nucleosomes at the transcription termination ends (TTEs, Figure 1b). These data are consistent with previous lower resolution studies that show PARP1 enriched at +1 and +2 nucleosomes of heat-shock genes [29, 30] as well as our recent high-resolution analyses of PARP1 binding in human cells [28]. Based on this observation, we further quantified the relationship between gene expression and PARP1 interaction with promoters, by calculating the Pearson correlation between gene expression and PARP1-nuc-ChIP-seq read depth across −50 to +500 bp surrounding annotated promoter regions. PARP1 association correlates positively with gene expression (Pearson correlation *R*=0.427; *P=*2.2e-16). Performing the same analyses at the ends of genes showed a very weak/no correlation with gene expression levels (*R*=0.0196; *P*=0.025).

### PARP1 associates with exonic nucleosomes

**Figure 2:**
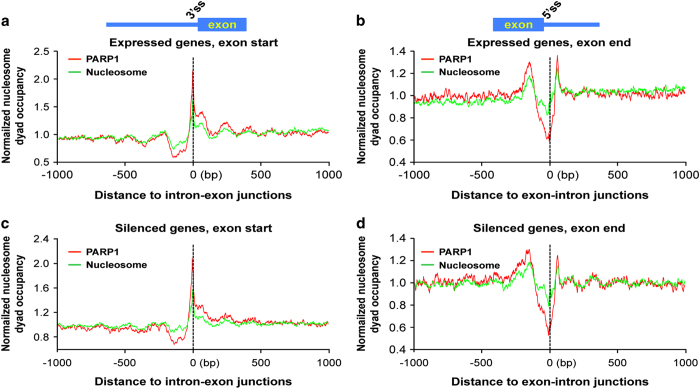
PARP1 demarcates exons and is enriched at intron/exon and exon/intron boundaries irrespective of the transcriptional state of the genes. For this calculation, intron-containing genes were used and curves were normalized by genome-wide average. Dyad density of PARP1 nucleosomes at (**a**) start of exons (intron/exon) and (**b**) end of exons (exon/intron) of active genes, respectively. Dyad density of PARP1 nucleosomes at (**c**) start of exons (intron/exon) and (**d**) end of exons (exon/intron) of inactive or silent genes, respectively. To control for the effect of nucleosomes, dyad density scores are normalized by the average density over the genome (see Supplementary Figure S1).

While profiling PARP1 genomic location, we observed PARP1-nucleosome signals at exon–intron boundaries. To test whether the binding of PARP1 in coding regions was related to the chromatin architecture around exon/intron junctions in *Drosophila*, a fine chromatin structure map of PARP1 occupancy across the coding regions of active genes and silent genes was generated (Figure 2). These analyses showed that PARP1 associated with nucleosomes within exons, irrespective of the transcriptional status of the genes (Pearson correlation *R*=0.325; *P=*1.5e-16) (Figure 2a–d). We next addressed whether the sharp peak of PARP1-bound nucleosomes observed at the exon/intron and intron/exon boundaries was a special feature of PARP1 chromatin structure at these regions. We compared the profiles of total nucleosome occupancy with those of PARP1-bound nucleosomes at the intron–exon boundaries (Figure 2a and c) and exon–intron boundaries (Figure 2b and d). We observed a difference in both profiles: higher peak in the PARP1-bound nucleosome profile with a deeper valley past these peaks than with total nucleosome profiles. From these analyses, we concluded the observed peaks in the PARP1-nuc-seq data are not solely the consequence of increased nucleosome occupancy in this region, but an enrichment of PARP1-bound nucleosomes at these regions.

**Figure 3:**
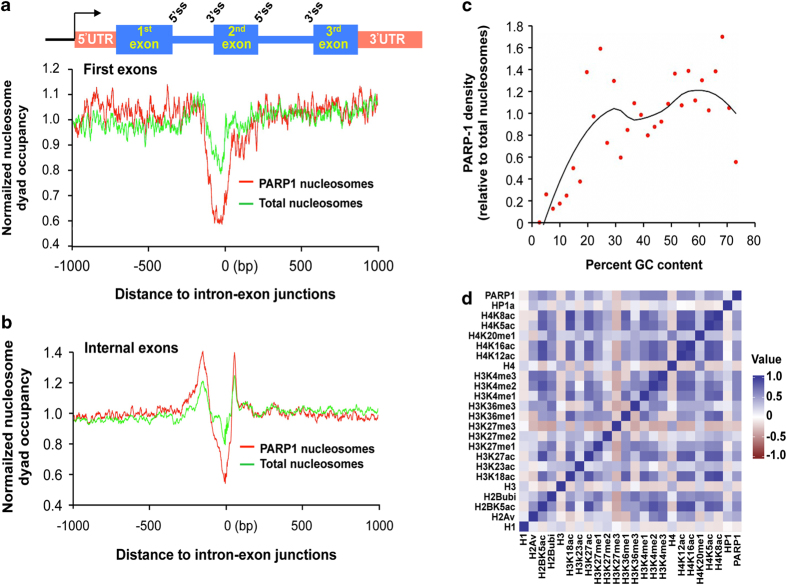
Characteristic features of PARP1-bound nucleosomes. (**a**) PARP1-bound nucleosomes (red curve) are not enriched at first exons boundaries but are (**b**) highly enriched at internal exons (tag densities were measured at ±1000 bp), even if the effect of total nucleosome (green curve) is subtracted. For this calculation only intron-containing genes were used. (**c**) PARP1 associates with GC-rich nucleosomes. Plot shows PARP1-bound nucleosome density relative to total nucleosome density as a function of GC content. The black curve is the fitted smoothing line by loess method (local regression) using locally weighted polynomial regression analyses. (**d**) Heatmap showing that PARP1 binding overlaps with chromatin regions occupied by specific histone modifications. Pearson correlations of PARP1 and the following epigenetic marks (all with *P*-values<10^10^).

To differentiate the observed effect of PARP1 at TSSs with those at the internal exons, we asked if there was a difference between PARP1-bound nucleosomes at the first exon/intron boundaries compared with PARP1-bound nucleosomes at internal exon/intron boundaries. To address this question, PARP1-bound nucleosome footprints centered on the 5’ sites of all exons were constructed. This analysis showed low levels of PARP1-bound nucleosomes at the ends of first exons (excluding ±250 bp surrounding annotated TSSs) (Figure 3a), whereas PARP1-bound nucleosomes were abundantly positioned at the start of internal exons (Figure 3b). To ensure that nuc-ChIP – seq technique did indeed pull down PARP1 targets, chromatin was digested with different amounts of MNase, resulting in varying lengths of chromatin (Supplementary Figure S2A). ChIP-qPCR (quantitative real-time polymerase chain reaction) was performed to validate PARP1 binding at target and non-target genes (Supplementary Figure S2B–D). The results show that PARP1-nucleosome binding is specific validating the nuc-ChIP-seq results (PARP1-nuc-ChIP profiles are shown in Supplementary Figure S2E). Analyses of gene ontology by the Database for Annotation, Visualization and Integrated Discovery (DAVID) Bioinformatics Resources (version 6.7) [31] of the genes bound at the internal exon/intron boundaries by PARP1 showed that these genes were involved in several important gene regulatory processes (Supplementary Figure S2F and Supplementary Table S1).

### PARP1 associates with GC-rich nucleosomes and with active histone modifications

Previous work demonstrates that the preference for nucleosome positioning over exons is influenced by nucleotide sequence composition. Across eukaryotes, exon sequences tend to have elevated GC content compared with flanking introns [7, 17, 20, 32]. As higher GC content confers stability to exonic nucleosomes [15, 33], we sought to determine whether PARP1-bound nucleosomes are particularly GC-rich. We found that as the GC percentile increases, more PARP1 reads (compared with total nucleosomes) were mapped, indicating that PARP1-associated nucleosomes at the internal exon/intron boundaries are, on average, GC-rich (Figure 3c). GC-rich nucleosomes have been suggested to bind to weak splice sites, slowing down the rate of RNAPII elongation [20, 34], which might promote co-transcriptional splicing.

We next investigated the association of PARP1 with specific histone modifications. We downloaded a large set of histone modification data on S2 *Drosophila* cell line from the modENCODE project [35]. Analyses of our PARP1-nuc-ChIP-seq results (PARP1 binding) showed an overlap of PARP1-binding with several active histone PTMs ChIP-seq data (Figure 3d) such as H3K4me3, H3K36me3, H4K16ac but not with the repressive histone PTM H3K27me3 nor H1.

### Global ASEs mediated by PARP1 and its PARylation activity

As PARP1 is associated with nucleosomes at exon boundaries, we hypothesized that PARP1-associated chromatin structure might function in alternative splicing. To test the functionality of PARP1 in alternative splicing, we used small interfering (siRNA) to knockdown PARP1 in S2 *Drosophila* cells (compared with cells treated with LacZ control siRNA). PARP1-knockdown efficiency was determined at both protein and mRNA levels (Supplementary Figure S3A and B). As previous studies have shown that PARylation of proteins within the spliceosomal complex is important for their activity [36, 37], we also asked whether the observed effects on alternative splicing were dependent on PARP1’s PARylation activity by inhibiting PARylation using PJ-34 [29, 38] (Supplementary Figure S3C).

To address the global impact of PARP1 on alternative splicing, we isolated total RNA in two biological replicates from control (non-treated), PARP1 siRNA- and PJ-34-treated cells. Sequencing of these RNAs on an Illumina HiSeq 2500 yielded >56 million 100-bp paired-end RNA-seq reads. First, we aligned reads to the entire gene body of PARP1 and confirmed a reduction in PARP1 expression of ~30% (*P*<10^−3^). This result is consistent with the quantitative reverse-transcription PCR (qRT-PCR) data (Supplementary Figure S3B), and confirms PARP1 depletion after PARP1 siRNA treatment (Supplementary Table S2). We next used these RNA-seq data sets (control, PARP1 KD and PARylation inhibited) to assess whether these treatments resulted in changes in (i) gene expression and (ii) alternative splicing.


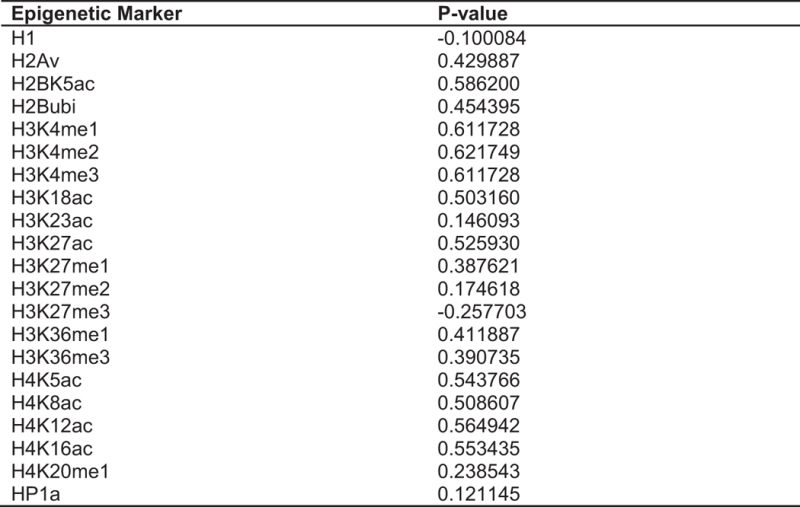


#### Gene expression patterns mediated by PARP1 and PARylation

Using the Tuxedo protocol for RNA-seq analysis [39], PARP1 depletion resulted in ~849 differentially expressed genes (DEGs) (*P*⩽0.05), of which 210 were upregulated (Supplementary Table S2C) and 639 were downregulated (Supplementary Table S2D). We used categoryCompare [40] to determine the function of these DEGs (Supplementary Figure S4A). Genes upregulated after PARP1 knockdown are mainly involved in nucleotide metabolism, ATP synthesis and spindle (centromere) formation, whereas those downregulated genes are involved in differentiation, growth, migration and development (Supplementary Table S2E). Inhibition of PARylation affected the expression of 1281 genes; 974 of these were upregulated (Supplementary Table S2A) and 307 were downregulated (Supplementary Table S2B) (*P*⩽0.05). Interestingly, the top category of genes upregulated after PARylation inhibition are involved in the spliceosome and in RNA polymerase-processing pathways, whereas downregulated genes are involved in neuronal differentiation, cell death and immune response (Supplementary Table S2F). Comparison of the DEGs mediated by PARP1 knockdown and PARylation inhibition reveals 182 genes common to both (Supplementary Figure S4A and B) with no common directionality in the gene expression profiles. Changes in gene expression were validated using qRT-PCR, with 8 out of 10 genes (90%) in accordance with RNA-seq data (Supplementary Figure S4C).

#### Alternative splicing patterns mediated by PARP1 and PARylation

**Figure 4:**
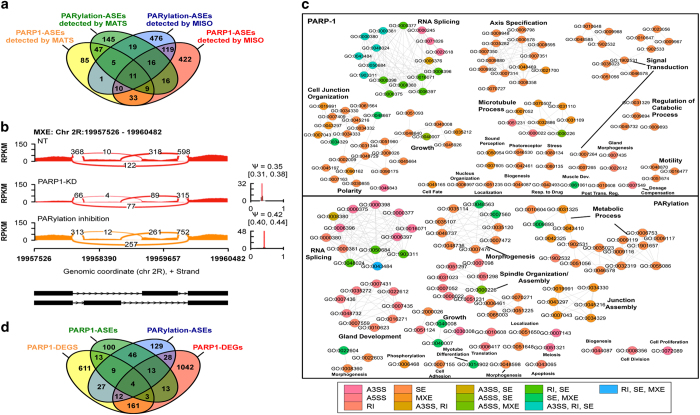
Global gene regulatory events mediated by PARP1 and its PARylation activity. (**a**) Four-way Venn diagram showing the different types of ASEs that are detected by either MATS or MISO detected by both methods. (**b**) Sashimi plots showing example of ASEs mediated by PARP1 and PARylation. RNA-seq read densities supporting ASEs and the estimated confidence levels are shown in the figure. (**c**) Visualization of the Gene Ontology Biological Process (BP) categories of PARP1 and PARylation-mediated ASEs. Circles are shaded based on types of ASEs as indicated on the legend. (**d**) Four-way Venn diagram summarizes the number of shared proteins in each combination of the four groups. Yellow and green: PARP1-mediated DEGs and ASEs, respectively; purple and red: PARylation-mediated DEGs and ASEs, respectively (*P*<0.01, by Student’s *t*-test). Numbers depicted in the intersections between circles represent the numbers of genes that are commonly regulated in two, three, or four conditions.

We asked next if PARP1 or PARylation controls the expression patterns of alternative transcript isoforms at the transcriptome level. MATS (multivariate analysis of transcript splicing) [41] and MISO (mixture of isoforms) [42] were used to estimate expression levels of different mRNA isoforms (Supplementary Figure S5A). Both methods use a Bayesian approach to calculate the ASEs between two samples; however, MATS measures ASEs, whereas MISO determines both isoform and event-inclusion level [43]. We used both methods to identify targets for which PARP1 or PARylation modulates the following pre-mRNA processing event subclasses: (i) mutually exclusive exons, (ii) cassette exons (skipped exons (SEs) according to MATs analyses), (iii) alternative 3′ and 5′ splice sites and (iv) retained introns (Supplementary Figure S5A). Some events were commonly detected only by MISO (Supplementary Table S3), whereas others were detected by both methods (Figure 4a and d and Supplementary Figure S5A; Supplementary Table S4), indicating the validity of these events. MISO (ΔΨ of 0.2 and Bayes factor ⩾10) detected a total of 1540 events (4.5% of total MISO-detectable ASEs) regulated by PARP1 independent of its catalytic activity and 745 events (2% of total MISO-detectable ASEs) regulated through the PARylation activity of PARP1. Likewise, MATS at *P*<0.005, identified 135 events (3% of total MATS-detectable ASEs) as regulated by PARP1 and 290 events (4% of MATS-detectable ASEs) as regulated through the PARylation activity of PARP1 (Figure 4a). Examples of ASEs determined by MISO are illustrated using Sashimi plots (Figure 4b and Supplementary Figure S5B). Overall, these results show that irrespective of the model used (MISO or MATS), PARP1 and PARylation have a broad role in regulating mRNA processing events in *Drosophila* S2 cells.

At last, we again performed categoryCompare analysis [40] to examine whether transcripts modified by PARP1/PARylation were enriched for particular functional categories (Figure 4c). The top category found to be affected by PARP1 depletion and inhibition of the PARylation activity of PARP1 were genes involved in RNA splicing, with many core and regulatory components (including members of the SR and hnRNP families). We conclude from these results that PARP1 and PARylation preferentially affect transcripts encoding protein products involved in RNA and protein-processing pathways (Figure 4c and Supplementary Table S3). Though, PARP1 depletion and PARylation inhibition affected the expression of several genes (Supplementary Figure S4A), 87% of the genes regulated by PARP1 at the splicing level were not affected in their level of mRNA expression (Supplementary Table S2). Of note, depletion of PARP1 did not substantially affect the expression of SFs (Supplementary Table S2C and D), though PARylation did affect the expression of SFs (Supplementary Table S2A and B). This suggested that there is a possible difference in the molecular mechanisms affected by PARP1 from its enzymatic activity.

We also analyzed the overlap between PARP1/PARylation RNA-seq data and PARP1-ChIP-seq data to determine the PARP1-bound genes that undergo PARP1/PARylation-mediated ASE changes. We observed an ~23–33% commonality, respectively, in these data sets (Supplementary Table S4). Given the technical differences in protocols and the inherent loss of information in threshold and significance threshold calls, this number of genes is likely a conservative estimate. Next, we identified genes affected at both their expression and splicing levels by PARP1 siRNA compared with inhibition of PARylation. Surprisingly, although these analyses revealed some commonalities, the profiles were largely different (Figure 4d). For instance, PARP1 and PARylation regulated only four genes in common at both gene expression and splicing levels: *CG40178, CG40191, ASCL* (Acyl-CoA synthetase long-chain) and PARP1 itself.

At the gene expression level, genes shared by the PARP1 and PARylation function in several pathways, including neuron differentiation, cell death and immune response. Intriguingly, at the splicing level, PARP1 and PARylation commonly regulated 72 genes as determined by MATS, of which many are involved in splicing (Figure 4a and c). These results are consistent with two possible modes of PARP1 function in alternative splicing that are not mutually exclusive: (1) as a structural chromatin protein, PARP1 can directly affect alternative splicing through association with chromatin structures that affect RNA polymerase elongation and/or act as an adapter for RNA and SF binding; and (2) through PARylation of SFs PARP1 can indirectly affect splicing by regulating the activity/expression of spliceosomal proteins (Figure 4).

#### Validation of ASEs regulated by PARP1 and PARylation

**Figure 5:**
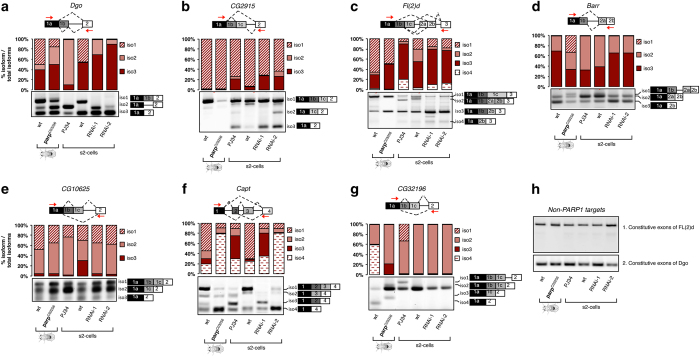
PARP1 regulates alternative splicing. (**a**–**g**) Measurements of ASEs at alternative exons. (**h**) Measured gene expression of constitutive exons. Representative gel images of the effect of PARP1 and PARylation on ASEs. Total RNA from wt and *Parp* ^*C03256*^ flies were tested for changes in ASEs (lanes 1 and 2). S2 cells treated with PJ34 (lane 3), S2 cells treated with (i) LacZ non-targeting siRNA (lane 4), (ii) PARP1 siRNA1, (iii) PARP1 siRNA2 (KD1 and KD2, lanes 5 and 6, respectively). Bar charts represent the alternative arbitrary units from qRT-PCR measured as a rate of alternative exon included over the sum of all the alternative exons calculated from the mean intensities (*n*⩾3 biological replicates, ±S.D.; *P*<0.05 with Student’s *t-*test; see Supplementary Figures S2 and 3). Black and white boxes represent constitutive exons 5′ and 3′ to the alternative exons (gray boxes), respectively. Red arrows depict locations of primers.

We used semi-qRT-PCR to validate the changes in alternative splicing observed in RNA-seq analyses after PARP1 KD and PARylation inhibition. For these analyses, we chose representative genes based on the ChIP-seq data showing PARP1-binding at internal exon–intron boundaries of target genes as well as RNA-seq experiments, showing PARP1-/PARylation-mediated ASEs at these genes. These splicing events fall into several classes: (i) alternative 5′ splice site (SS) usage, (ii) alternative 3′ SS usage and (iii) exon skipping. We confirmed that knockdown of PARP1 resulted in changes in ASEs at these genes (Figure 5, lane 5: RNAi-1). A second siRNA targeting a distinct sequence within the PARP1 gene, KD2, was used to corroborate that the observed changes in alternative splicing was due to the knockdown of PARP1 and not specific to the sequence of the PARP1 siRNA (Figure 5, lane 6: RNAi-2).

We also tested and validated the effect of the PARylation activity of PARP1 in S2 cells on alternative splicing of these genes (Figure 5, Lane 3). We further confirmed the effect of PARylation on alternative splicing in a fly model, *PARP* ^*C03256*^ that expresses a short isoform of the PARP1 protein lacking the first zinc finger, with reduced PARylation activity [44]. Indeed, comparing splice isoforms between wt and *PARP* ^*C03256*^ mutant flies showed changes in ASEs (Figure 5, lane 1 and 2). The observed splicing patterns were further validated quantitatively using quantitative real-time PCR at specific alternative exons (Supplementary Figure S6A and B). In all experimental conditions tested (PARP knockdown, PJ34-treated and *PARP* ^*C03256*^) compared with wild-type cells or flies, no changes in the expression at constitutive exons were observed (Figure 5h).

### PARP1 is recruited to chromatin and pre-mRNA

**Figure 6:**
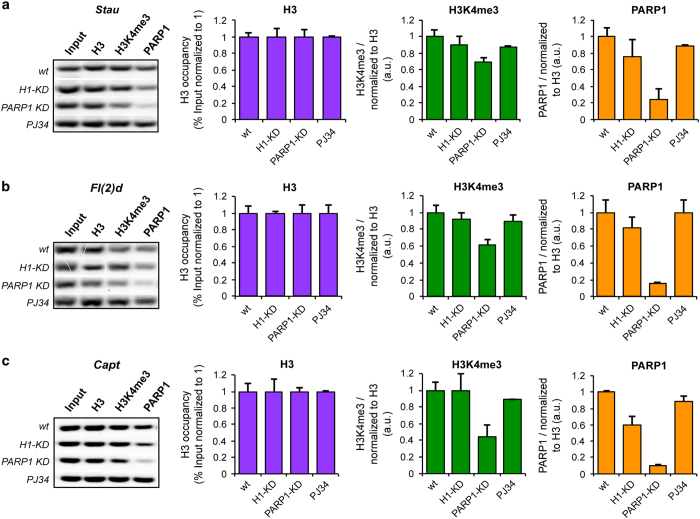
Co-occupancy of PARP1 and H3K4me3 at PARP1-target exons. S2 cells were transfected with siRNA targeting lacZ (NT) and PARP1 (PARP1 KD). ChIP-qPCR analysis of PARP1, nucleosome density (as measured by H3) and H3K4me3 occupancies at PARP1-target exons in (**a**) *Stau*, (**b**) *Fl(2)d* and (**c**) *Capt1* genes were analyzed in NT, PARP1 KD and PJ34-treated S2 cells. As proof of the specificity of the observed effect, these same factors were measured in Histone H1 Knockdown cells (H1 KD). Representative inverted agarose gel images of qPCR products stained with Gelstar are shown (far left). Bar graphs show ChIP-qPCR (quantitative real-time PCR) analyses normalized to IgG control of the measured occupancies. Antibodies used are indicated above each graph; results are represented as mean plus s.e.m. (*n*⩾3; Student's *t*-test, *P*<0.05). Primers that target the alternative exons as in Supplementary Figure S5B were used in ChIP PARP1, H3K4me4 co-occupancy assays.

Based on the findings above, we considered three possible recruitment mechanisms for PARP1 in splicing:

First, we asked whether the splicing effects we observed after PARP1 knockdown or PARylation inhibition were due to changes in nucleosome occupancy over these exons. For this, we carried out ChIP-qPCR analyses to measure nucleosome and PARP1 occupancy at the exons of selected genes. Knockdown of PARP1 (PARP1 KD) resulted in depletion of PARP1 occupancy at these experimental exons – *Stau* (Figure 6a); *Fl(2)d* (Figure 6b); *Capt* (Figure 6c) with no measurable change in nucleosome density, as measured by histone H3 occupancy (Figure 6, Lane 2). Contrary to PARP1 KD, PARylation inhibition had no effect on nucleosome density (H3 occupancy) or PARP1 occupancy (Figure 6a–c, Lane 2 and 4), corroborating previous results [45].

Second, we asked if at these exons, PARP1 associates with specific histone modifications that have been implicated in splicing. In these studies, we focused on H3K4me3 for several reasons: (1) H3K4me3 is proposed to facilitate SF loading [10], (2) we show both in human cells [46, 47] and S2 *Drosophila* cells (Figure 3d) that PARP1 binding correlates positively with H3K4me3 presence (in S2 *Drosophila* cells with a Pearson correlation *R*=0.6215; *P*<10^−10^). ChIP-qPCR analyses confirmed the co-presence of H3K4me3 and PARP1 at the nucleosomes of these PARP1-target exons (Figure 6a–c, Lane 3 and 4). In the absence of PARP1 (PARP KD), a concomitant reduction (~60%) of H3K4me3 occupancy at these target exons was observed (Figure 6a–c), confirming previous studies of a role of PARP1 in H3K4me3 deposition [46]. We therefore hypothesized that this reduction is specific for PARP1 knockdown as knockdown of Histone H1, a protein that competes for nucleosomal binding with PARP1 [27], had no significant effect on H3K4me3 or PARP1 occupancies at these exonic sites (Figure 6, Lane 3 and 4). However, at other PARP1-target regions, no H3K4me3 was found (Supplementary Figure S6C), implying that other factors or histone modifications might also be important for PARP1-nucleosome association. On the other hand, compared with PARP1 KD, no reduction in H3K4me3 occupancy (Figure 6, Lane 3) was observed in PJ34-treated cells as previously reported [46]. We believe the difference could be due to length of PJ34 treatment and/or cell type (S2 cells with one PARP1 and MCF7 cells with several PARP1s). Taken together, these results support our findings of the differential splicing outcomes in PARP1 KD and PARylation-inhibited cells. And, as with the case of PARP1’s function in transcription, the physical presence of PARP1 is critical in some molecular pathways, possibly involving the chromatin structure, whereas PARylation is important in others [27, 44, 48].

**Figure 7:**
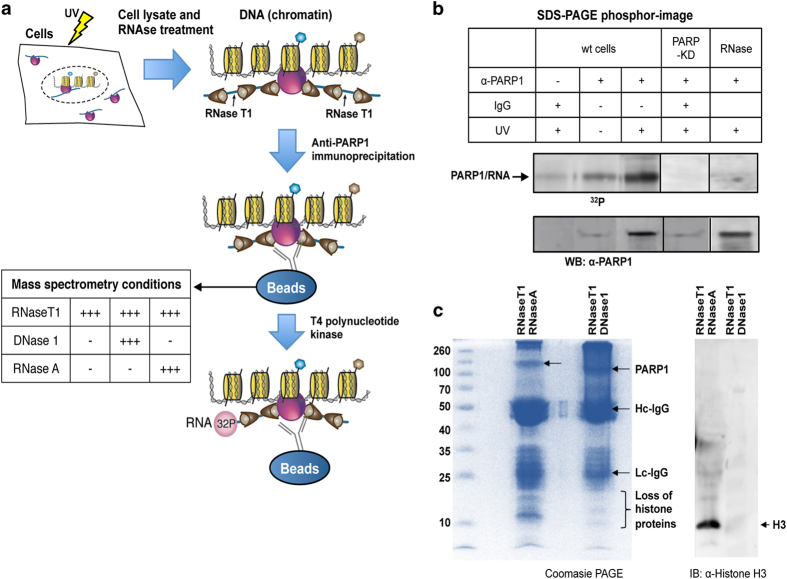
Identification of PARP1 interactome *in vivo*. PARP1 is covalently cross-linked to nascent RNAs using 365 nm iUV light and 4-thiouridine. PARP1-bound nascent RNAs are immunoprecipitated using PARP1 antibody and then purified under stringent conditions. (**a**) PAR-CLIP procedure. (**b**) Radiolabeled PARP1-bound RNAs are blotted onto nitrocellulose, released by proteinase K and analyzed on a phosphoimager. The same blot was probed with anti-PARP1 antibody confirming PARP1-RNA binding. Knockdown of PARP1 or stringent RNase treatment of immunoprecipitated samples eliminated the PARP1-RNA band. Furthermore, protein samples resulting from PAR-CLIP experiments were split into three aliquots (i) no further treatment; (ii) stringent DNase1 treatment; (iii) stringent RNase A. These samples were subjected to mass spectrometry (Supplementary Table S5). (**c**) Proteins released from stringent RNase A and DNase1 digests were analyzed on a coomassie-stained gel and also probed with anti-H3 antibody to show depletion of histones.

Third, we hypothesized that PARP1 might act as an adapter bringing RNA to specific regions of chromatin. To test this hypothesis, we first sought to determine whether PARP1 binds RNA *in vivo*. To this end, PAR-CLIP (photoactivatable cross-linking and immunoprecipitation) protocol [49] (Figure 7a) was used and PARP1-associated RNAs were immunoprecipitated with a PARP1 antibody (Figure 7a and b). As spliceosome dynamics and composition are very similar between *Drosophila* and humans [50], and most known human-SFs have orthologues in flies [51], experiments were performed in both S2 and HeLa cells. Complexes representing the expected molecular weight of a single molecule of PARP1 bound to its target RNAs were observed in both cell types. This band was eliminated in stringent RNase A treatment as well as in PARP1 KD cells (Figure 7b and Supplementary Figure S7A). Our data from three independent experiments show direct *in vivo* binding of PARP1 to RNA, supporting previous studies that showed or postulated the binding of PARP1 to RNA and RNA binding proteins [52–54]. In effect, the binding of PARP1 to mRNA at some target exons (Figure 5) was validated after PAR-CLIP in both cell types using RT-PCR (Supplementary Figure S7B). Our inability to validate CAPT/CAP mRNA binding could be due to the differences in conditions used in the different methods (nuc-ChIP-seq *versus* PAR-CLIP-qRT-PCR).

As PARP1 is a known chromatin-binding protein, we tested the possibility that PARP1 binds both RNA and chromatin at the same time. Following the PAR-CLIP experiment and after PARP-IP − although the PARP1:RNA complexes were still on beads − samples were subjected to polyacrylamide gel electrophoresis (PAGE) analyses. Gel pieces were excised, trypsin-digested and used for tandem mass spectrometry analyses to determine the protein components. We filtered and retained only proteins with a stringent mascot level of >23, with more than four unique peptides and present in four independent experiments. We observed the presence of histones, other chromatin proteins and proteins involved in pre-mRNA regulation (Figure 7c and Supplementary Table S5). Based on these results, we believe that PARP1 is in a complex with chromatin, RNA and SFs. Remarkably, the stringent RNase A or DNase I digest of PAR-CLIP samples resulted in the a significant number of RNA or DNA (chromatin) proteins, respectively (Figure 7 and Supplementary Table S5).

**Figure 8:**
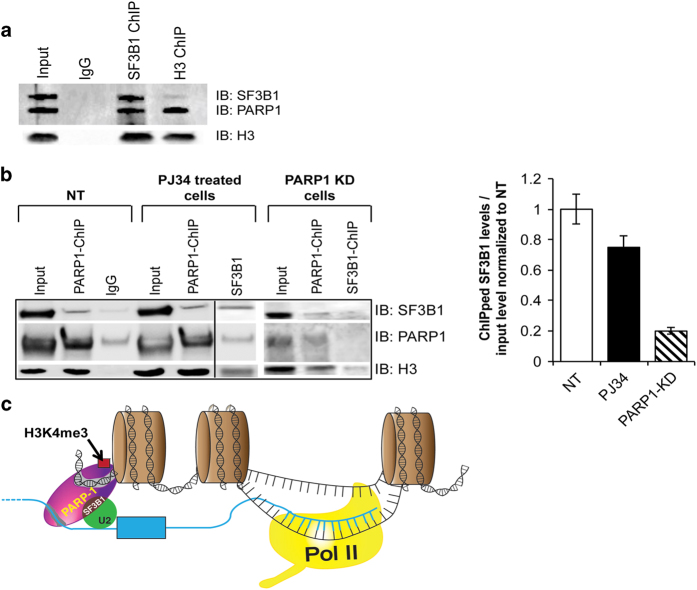
PARP1 regulation of co-transcriptional splicing. (**a**) PARP1 and SF3B1 bind to the same nucleosomes. SF3B1 (U2 snRNP) ChIP shows that most SF3B1-bound nucleosomes (SF3B-IP) also bind PARP1. However, PARP1 binds other nucleosomes as indicated by H3 ChIP. Knockdown of PARP1 impaired the association of SF3B1 to nucleosomes, whereas inhibition of PARylation had no significant effect. (**b**) ChIP experiments showing co-occupancy of PARP1 and SF3B1 in HeLa cells. Knockdown of PARP1 resulted in reduction of SF3B1-nucleosome association, whereas PARylation inhibition (PJ34 treatment had no such effect). The results (bar graph) are represented as mean plus s.e.m. (*n*⩾3; Student's *t*-test, *P<0.05). (**c**) Model of PARP1 in mediating co-transcriptional splicing. PARP1 binds to specific nucleosomes at exons (specified by specific histone PTMs, for example, H3K4me3) and also binds to the nascent pre-mRNA and recruits SF3B1 a U2 component. U2 binds to the branch-point recognized by the splicing machinery, allowing PARP1 to influence exon recognition. RNA polymerase II generating the nascent pre-mRNA is shown on the right.

Fourth, PARylation has been proposed to assist in the opening of chromatin structure and to increase access of factors to DNA [29, 30, 55]. Given that PARP1 binds chromatin and mRNA (Figure 7) and given that PARylation activates SFs [37, 44, 56] we speculated that PARP1 might be recruiting SFs. For this purpose, we analyzed the association of PARP1 and the SF 3B subunit 1 (SF3B1), a component of the U2 snRNPs. Owing to the availability of ChIP-grade antibodies, these experiments were carried out in human HeLa cells. NChIP experiments using antibodies specific for SF3B1 were performed and probed for the presence of PARP1. Most SF3B1-bound nucleosomes also contained bound PARP1 as exemplified with SF3B1-ChIP (Figure 8a, Lane 3). Furthermore, in H3-pulldowns, we observed the presence of some SF3B1, implying that SF3B1 binds only a small subset of all nucleosomes (Figure 8a, Lane 4). PARP1, on the other hand, binds to more nucleosomes, possibly because of its role in other chromatin gene regulatory programs, not necessarily linked to SF3B1. To provide additional evidence for a physical association between PARP1 and SFs, we fractionated HeLa nuclear extracts on a 10−30% glycerol gradient. We observed PARP1 co-sedimenting with SF3B1 (fractions 6–14 in Supplementary Figure S8), arguing for a stable association between PARP1 and SF3B1. These results are in line with previous studies showing spliceosomal complexes as part of PARP1 interactome [57] and vice versa: PARP1 as one of the interacting proteins in the spliceosomal complex [58]. As PARylation is known to affect protein complex stability [59, 60], we asked whether this PARP1-SF3B1 association requires PARylation. Though PJ34 treatment resulted in a significant decrease in global PARylation levels (Supplementary Figure S3C), this treatment did not abrogate PARP1-SF3B1-nucleosome association as measured by ChIP (Figure 8b). Knockdown of PARP1 resulted in less SF3B1 association with nucleosomes, implicating PARP1 in stabilizing the SF3B1-nucleosome complex (Figure 8b). Taken together, our results support the idea that a fraction of PARP1 is in a complex with spliceosomal components, possibly recruiting them to relevant sites important for splicing regulation.

## Discussion

The findings presented here reveal a functional role for PARP1 in the regulation of pre-mRNA splicing. In particular, we have shown that PARP1 binds to nucleosomes at exon/intron boundaries corresponding to specific splice sites. In addition, we demonstrate that knockdown of PARP1 or inhibition of its PARylation activity, leads to changes in specific alternative splicing patterns. Moreover, PARP1 and its PARylation activity have distinct effects on splicing with nucleosomal PARP1 causing direct changes in ASEs, whereas PARylation inhibition resulted in changes in gene expression of SFs, implying an indirect effect on splicing. Our findings thus reveal an important role for PARP1 in regulating alternative splicing, at both the gene-specific and global level. Importantly, we find that in addition to its well-established association with chromatin, PARP1 displays *in vivo* RNA binding and binds to SF3B1, a member of the U2 spliceosomal complex.

Our studies show also that the effects of PARP1 or PARylation inhibition are quite distinct from each other, with no specific direction in splicing modulation (exon inclusion or exon exclusion). Surprisingly, our analyses showed little overlap between ASEs modulated after PARP1 knockdown and PARylation inhibition. We hypothesize that these differences are due to several possible reasons:

First, PARP1 knockdown results in a general reduction in PARP1 occupancy over PARP1-target exons, whereas PARylation inhibition does not change PARP1 occupancy (Figure 6). In fact, we observed a slight reproducible increase in PARP1 occupancy after PARylation inhibition, though more studies will be needed to validate this finding. However, these results are in line with previous studies showing that extensive PARylation of PARP1 inhibits its nucleosome binding [61–63]. On the other hand, it is plausible that lack of PARylation results in less-PARP1 degradation, as PARylation negatively regulates certain proteins by dissociating protein complexes or by promoting their ubiquitination and their proteasomal degradation [59, 60].

Second, our studies show that with PARP1 knockdown, PARP1 is absent at these exons with a concomitant decrease in H3K4me3, whereas with PARylation inhibition, PARP1 and H3K4me3 are present. If PARP1 were to function by regulating the deposition of H3K4me3 [46], a histone modification implicated in splicing, there would be a difference between the splicing outcomes between PARP1 KD and PARylation inhibition.

Third, PARylation inhibition had no effect on PARP1-SF3B1-nucleosome binding (Supplementary Figure S8A). A similar situation has been shown during transcription, where PARP1 is essential for recruiting the transcription machinery, such as the mediator complex or co-regulators (for example, p300), and its catalytic activity is not required in these processes [64, 65].

Our studies showing differential effects on ASEs mediated during PARP1 KD and PARylation inhibition is thought to be a consequence of the absence of PARP in PARP1 knockdown, whereas in PARylation inhibition, PARP1 is still present and can effect changes in ASE. This finding supports distinct roles of PARP1 and PARylation in splicing regulation; a direct effect by PARP1 produced by the presence of the PARP1 protein and an indirect effect produced by PARylation. Indeed PARylation has been shown to have key roles in transcription regulation in some contexts [44, 46, 66], whereas being dispensable in others [67, 68]. A recent study by Muthurajan *et al*. [48]. shows that PAR on PARP1 switches PARP1 from a chromatin architectural protein to a histone chaperone and nucleosome assembly factor, demonstrating differential functional outcomes between PARP1 as a protein *per se* and PARP1’s catalytic activity. In addition, though the *Drosophila* genome encodes only one PARP1 protein, it also encodes a tankyrase (PARP5 in humans) with a PARylation activity, therefore the differences in PARP1 knockdown effect *versus* PARylation inhibition on splicing, could also be masked by functional redundancy.

Finally, the co-presence of PARP1 and H3K4me3, may point to the specificity of PARP1 binding. However, positive correlations genome-wide of PARP1-chromatin-binding with chromatin regions containing other histone modifications such as H3K36me3 and H2Av (Figure 2c) that have been implicated in splicing (reviewed in ref. 69) were also observed. It is therefore possible that other histone modifications might also be important in targeting PARP1 to specific regions of the genome. It is therefore possible that PARP1 recognizes specific histone modifications to regulate alternative splicing decisions. Indeed, it has been hypothesized that adapter proteins recognize specific histone modifications and recruit SFs close to chromatin and thus aid in co-transcriptional splicing. The activities of adapter proteins range from activation and repression of transcription, chromatin remodeling, or splicing efficiency as well as other activities [70]. It has been suggested that H3K4me3 serves to facilitate the competency of pre­mRNA maturation through the bridging of spliceosomal components [10]. PARP1, therefore, might act as an adapter associating with H3K4me3 at exons and aiding to bridge SFs to functional sites on chromatin.

We propose the following model (Figure 8c): PARP1 binds to nucleosomes containing H3K4me3 at exons as well as sites on nascent RNA, and through its association with U2 snRNP regulates usage of alternative exons. In this scenario, PARP1 not only acts as an adapter, ensuring that nascent pre-mRNAs are held close to chromatin [71], but also marks the exons both on chromatin and the nascent pre-mRNAs. Our data (Figure 1) showing PARP1 presence at exons and our ability to amplify some of the PARP1-bound exons from PARP1-bound RNAs (Supplementary Figure S6B) support this idea. Furthermore, PARP1 bound at these exonic sites on pre-mRNA is then able to recruit SFs to these regions. Several observations support this possibility. First, our CLIP-mass spectrometry data show that PARP1 is in a complex with chromatin, and associate with RNA and SFs (Figures 7 and 8a and b and Supplementary Figure S7A). Also PARP1 knockdown results in impaired SF3B1-nucleosomal recruitment (Figure 8b). Our studies suggest that PARP1 adds another layer of complexity to chromatin modulation in co-transcriptional splicing, and support a model in which PARP1-containing complexes regulate gene expression at both transcription initiation and pre-mRNA-splicing levels. Future studies are important to decipher the mechanism of PARP1 in RNA biogenesis.

## Materials and Methods

### S2 and HeLa cell culture

*Drosophila melanogaster* S2-DRSC cells (obtained from the *Drosophila* Genomics Resource Center) were cultured in Schneider’s *Drosophila* medium (Life Technologies, Grand Island, NY, USA) supplemented with 10% fetal calf serum (Hyclone, Logan, UT, USA). HeLa cells were obtained from the American Type Culture Collection (Rockville, MD, USA) and cultured Dulbecco’s modified Eagle’s medium containing 1 mM sodium pyruvate, 0.1 mM non-essential amino acid, 10% fetal calf serum, 100 U/ml penicillin and 100 μg/ml streptomycin at 37 °C in a humidified environment containing 5% CO_2_ and 95% air.

### Drosophila strains

All Drosophila strains were kept on standard media at 25 °C. wild-type Oregon R and PARP^C03256^ mutant flies [44] were obtained from Professor Tulin. All experiments used cells were experimental samples and controls were growth time and cell-density matched.

### ChIP of PARP1-bound nucleosomes

Chromatin fixation and immunoprecipitation were performed essentially as described by [72]. In brief, 1×10^7^ cells were resuspended in PBS and fixed with 1% formaldehyde for 10 min. Next, cells were washed and pelled cells were resuspended in lysis buffer (1% SDS, 10 mM EDTA, protease inhibitors, 50 mM Tris–HCl, (pH 8)) for 5 min on ice. Resulting nuclei were pelleted and washed with MNase buffer (10 mM Tris (pH 7.4), 15 mM NaCl, 60 mM KCl, 0.15 mM spermine, 0.5 mM spermidine, 2 mM CaCl_2_). Chromatin was subjected to micrococcal nuclease (MNase) digestion, with varying concentrations of MNase concentration, in MNase buffer at RT to yield nucleosomal fragments. The addition of 25 mM EDTA and 0.2% SDS stopped the reactions. Cellular debris was pelleted and the supernatant recovered. Lysates were diluted 1:10 in ChIP dilution buffer (0.01% SDS, 1.1% Triton X-100, 1.2 mM EDTA, 167 mM NaCl, protease inhibitors, 16.7 mM Tris–HCl, (pH 8). Non-specific background was removed by incubating the MNase digested chromatin with Protein A/G dynabeads (Invitrogen, Carlsbad, CA, USA) overnight at 4 °C with rotation in the presence of BSA (250 μg ml^−1^). Precleared chromatin solutions were incubated overnight at 4 °C with rotation with antibodies against PARP1 (#39559, Active Motif, Carlsbad, CA, USA), H3 (ab1791, Abcam, Cambridge, MA, USA) and for control, immunoglobulin G (I8140; Sigma-Aldrich, St Louis, MO, USA). For quality control, 100 μl of the precleared chromatin was purified by QIAquick PCR Purification Kit (Qiagen, Germantown, MD, USA) and DNA fragment sizes were analyzed and confirmed to correspond to one to three nucleosome fragments. For the ChIP samples, the complex was washed and eluted, and immunoprecipitated material was purified using the QIAquick PCR Purification Kit (Qiagen). The purified DNA was analyzed by qPCR with respect to input using GelStar (Lonza, Walkersville, MD, USA)) and *Taq* DNA Polymerase (Invitrogen).

Quantitative real-time PCR was used to measure PARP1, nucleosome and H3K4me3 occupancy. ChIP-qPCR experiments were done with the following antibodies: PARP1, H3 and H3K4me3 to measure for PARP1, nucleosome and H3K4me3 occupancy according to ref 72. PCR signals from immunoprecipitated samples were normalized to input and in some cases to the total histone H3 control.

### Nuc-ChIP-seq and MNase-seq data analyses

Nuc-ChIP-seq was performed following the protocol described in our recent publication [28]. As no peak-calling program has gained consensus acceptance by the scientific community as the preferred tool for ChIP-Seq data analysis [73], we used two different methods to analyze the sequenced reads.

#### Method 1

Nucleosomes were defined as described in [74, 75]. To define nuc-ChIP- seq and MNase-seq centers based on the single-end reads we first estimated the average DNA insert length, *d*. We calculated the cross-correlation of the tag start positions between the reads on the Watson and Crick strands and identified the peaks at *d*=156 and *d*=150 for the PARP1-bound nucleosomes and total nucleosomes, respectively. Thus, the tags on the Watson and Crick strands were shifted by *d*/2 toward 3′ and 5′, respectively, so that the tag frequency on position *i* can reflect the probability of position *i* being the PARP1-bound nucleosome or total nucleosome centers. The relative abundance of PARP1-bound nucleosomes was estimated by counting the number of PARP1-ChIP-seq nucleosome centers within each region and dividing that by the total number of mapped reads. Custom scripts were used to calculate the nucleosome center as previously done [28, 74, 75].

#### Method 2

Model-based Analysis for ChIP-Seq (MACS) was used to identify peaks from the nuc-ChIP-Seq data as follows. For each replicate, a default *P*-value significance of 1×10^−5^ was defined as significant. ChIP-Seq peaks were detected using Macs2 [76, 77], with the broad option and a window size of 200 bp. Overlaps between each ChIP-Seq data peaks and PARP1 peaks were determined by using an overlap of at least 10% of the ChIP-Seq peak with the PARP1 peak.

Both methods yielded very similar results in spite of having different limitations: method 1 produces background noise and method 2 possibly eliminates PARP1 binding sites.

To visualize PARP1-bound nucleosome tag-density across the human chromosomes on the UCSC browser for hg19, ready-to-visualize bedgraph files were created using the HOMER package v3.13 [78]. In brief, aligned reads were extended to the average fragment size (109–160 bp) and read coverage on each base across the genome was calculated. Read coverage was then scaled to one million and normalized with the total number of reads. All publicly available data used for pairwise comparisons with our PARP1 data were processed in the same way. We also used total nucleosome (nucleosome-seq) data to normalize for background correction.’

### Native ChIP (NChIP) of SF3B1-bound nucleosomes

NChIP was performed similarly to nuc-ChIP described above albeit with no fixation step. In this protocol, antibodies used were SF3B1, PARP1 and H3 antibodies.

### HeLa cell extract fractionation

HeLa cell extract fractionation was done according to ref. 79. In brief, nuclear extract from HeLa cells was loaded onto a linear 4-ml 15–30% glycerol gradient prepared in G150 buffer (20 mM HEPES, pH 7.9, 150 mM NaCl, 1.5 mM MgCl_2_, 0.5 mM dithioerythritol). Gradient was centrifuged in a Beckman SW60Ti rotor at 35 000 rpm, at 4 °C for 15 h. Thereafter, the gradient was fractionated into 26 fractions of 150 μl each. In total, 30 μl aliquots from the fractionated samples were loaded onto 10% SDS–PAGE. To estimate the Svedberg (S) values, we used conalbumin (molecular weight 75 000 D; 5.4S), aldolase (15 800 D, 11.5S) and ferritin (440 000, 17S), all from GE healthcare, Piscataway, NJ, USA.

### siRNA production and treatment of cells

PCR product made against specific exons of PARP1 (siRNA1 for KD1) was obtained from the Lis laboratory - Cornell University, Ithaca, NY, USA [29]. PCR products made against the specific exons of PARP1 (siRNA2) and LacZ were obtained from the Drosophila RNAi Screening Center (FlyRNAi.org – the database of the *Drosophila* RNAi screening center: 2012 update) to produce double-stranded RNA (dsRNA) for PARP1 knockdown and non-targeting control LacZ. PCR products were further amplified and dsRNA was produced and purified according to the manufacturer – MEGAscript RNAi Kit (Life Technologies). RNAi treatments were as follows: S2 cells were treated with dsRNA, produced according to the Ambion MEGAscript manual. To achieve efficient RNAi knockdown (>30%), concentrations of ~10 nM of dsRNA (siRNA) were used. siRNA targeting the coding sequence of β-galactosidase (LacZ) was used as a non-specific control. SiRNAs against human PARP1 (ON-TARGET*plus*) from Thermo Scientific Dharmacon and transfection was performed as per the manufacturer using Dharmafect reagent 2.

### Inhibition of PARP1 in *D. melanogaster* cells

PJ34 was added to S2 cells in media, at final concentrations of 300nM and 10μM and incubated further for 10h at growth temperature [29, 38]. Cells were harvested for total RNAs extraction. Inhibition of PARylation was validated using Trevigen’s Pharmacodynamic Assay II (4520-096-K) according to manufacturer’s instructions.

### Antibodies

The following antibodies were used in this study: CHIP-grade PARP1 (Active Motif: 39559), CHIP-grade H3 antibody (Abcam: ab1791), CHIP-grade H3K4me3 antibody (Abcam: ab1791) and CHIP-grade custom-made SF3B1, which was a kind gift from the Stamm laboratory, University of Kentucky, Lexington, KY, USA.

### PAR-CLIP

PAR-CLIP was performed according to ref. 49. Samples were run on 8–20% PAGE gel (Invitrogen) and transferred on nitrocellulose membrane before visualization on a phosphoimager. Segments representing PARP1-bound RNAs were excised from membrane, purified and reverse transcribed into complementary DNA (cDNA) using qScript cDNA Supermix (Quanta Biosciences, Gaithersburg, MD USA).

### Analyses of gene expression data

*Drosophila* genome TSSs, transcription stop ends, exon/intron junction data and gene expression data were downloaded from http://genome.ucsc.edu/cgi-bin/hgTables and http://intermine.modencode.org, respectively, and processed as below:

### RNA-seq data analyses

A total of six fastq files were analyzed in this study, each representing a separate sample (two control; two PARP1 KD, and two PARylation). These fastq files were examined for quality control using FastQC [80]. Examination of the results of FastQC indicated that no further pre-processing was necessary. The RNA-seq reads were then processed using the Tuxedo suite [39]. The first step mapped reads to the Drosophila reference genome (dm3) using TopHat2 v2.0.10 [81] with the multiprocessor option –p 5 and the remaining parameters as the TopHat2 defaults, allowing at most two mismatches to the reference. In this analysis, we compared Control with PARP1 KD and PARylation. Transcripts were assembled using Cufflinks v2.1.1 [39]. Aligned reads were used as inputs into Cuffdiff2 [39] to detect DEGs, transcripts and ASEs along with their abundances, guided by the flyBase dm3 gtf track downloaded from the UCSC Genome Browser (http://genome.ucsc.edu). A min-alignment-count of seven reads was used as the threshold of the minimum number of reads that map to each transcript, and a *P*-value ⩽0.05 was used to determine differentially expressed (DE) events. FPKM (Fragments Per Kilobase per Million mapped reads) correlation analysis showed high correlation between the two control samples (Pearson correlation of *r*=0.85; *P=*3.14×10^−13^), between the two PARP1-knockdown samples (Pearson correlation of *r*=0.985; *P=*5.29×10^−21^) and between the two PARylation inhibition samples (Pearson correlation of *r*=0.980; *P=*9.37×10^−33^), indicating a very good transcript expression correlation between replica libraries.

#### ASEs using MATS

MATS [41] was used to measure ASEs. Results were generated for five events: SE, mutually exclusive exons, alternative 3′ splice sites, alternative 5′ splice sites and retained introns. The cutoff splicing default difference is 0.0001 for 0.01% difference. Thus, the valid cutoff used is 0⩽ cutoff <1. Second, AS events whose gene expression levels differed more than the given cutoff fold change between the two samples were filtered out. Valid: fold change>1.0. The default is 10000.0.

#### Alternative splicing analysis Using MISO

Alternative event annotations for *D. melanogaster* were downloaded from the MISO annotations page [42]. First, alternative event annotations were indexed using the index_gff module, which is part of the MISO toolkit. We ran MISO to get isoform expression estimates. In this step, Psi values were computed for each sample. We then made pairwise comparisons between samples to detect DE isoforms/events. In this step, Bayes factors and delta Psi values were computed between the samples. The last step was to filter DE events based on different criteria. Given a MISO Bayes factor comparison file for two-isoform events, events can be filtered based on their coverage or magnitude of change. We filtered our events based on the following parameters: (a) at least 1 inclusion read (–num-inc 1); (b) at least 1 exclusion read (–num-exc 1); (c) the sum of inclusion and exclusion reads is at least 10 (–num-sum-incexc10); (d) the Δ Ψ is at least 0.20 (–delta-psi 0.20); (e) the Bayes factor is at least 10 (–bayes-factor 10).

### Comparing RNA-seq PARP1/PARylation-mediated ASEs and PARP1 binding from nuc-ChIP-seq

We used Bowtie version 1.0.1 to map the read sequences to the Drosophila genome dm3. We then used MACS (version 1.4.2 to call peaks for each replicate (and combined) PARP1-binding site. We counted the number of peaks corresponding to each event, by determining the overlap between each peak file (PARP1 binding) with each event file (ASE). To determine whether there were PARP1-binding sites associated with the significant ASEs generated by MATS, we computed the number of peaks (per replicate and combined) that correspond to each event for each comparison.

### Liquid chromatography-electrospray ionization-tandem mass spectrometry analysis

After PAR-CLIP, samples on the beads were subjected to SDS loading buffer and PAGE electrophoresis as follows: one-third of IP samples were used directly for mass spectrometry to determine the protein complex associated with PARP1 RNA. The other third was subjected to stringent RNase A digest and the last third was subjected to stringent DNase1 digest. These latter samples were also used for (MS/MS) mass spectrometry analyses, followed by scanning with Mascot as previously reported [82]. Peptide matches that pass the filter associated with the strict False Discovery Rate (with target setting of 0.01) are assigned as high confidence. For MS/MS ion search, proteins with two or more high confidence peptides were considered unambiguous identifications without manual inspection. Proteins identified with one high confidence peptide were manually inspected and confirmed. To generate the list of proteins, we filtered the peptides retrieved from the database, leaving only those with individual Mascot scores above 23 and eliminating any consistent matches with IgG control sample as a background.

LC-MS/MS analysis was performed using an LTQ-Orbitrap mass spectrometer (Thermo Fisher Scientific, Waltham, MA, USA) coupled with an Eksigent Nanoflex cHiPLC system (Eksigent, Dublin, CA, USA) through a nano-electrospray ionization source. The peptide samples were separated with a reversed phase cHiPLC column (75×150 mm) at a flow rate of 300-nl/min. The mobile phase A was water with 0.1% (*v*/*v*) formic acid, whereas B was acetonitrile with 0.1% (*v*/*v*) formic acid. A 50-min gradient condition was applied: initial 3% mobile phase B was increased linearly to 50% in 24 min and further to 85% and 95% for 5 min each before it was decreased to 3% and re-equilibrated. The mass analysis method consisted of one segment with eight scan events. The first scan event was an Orbitrap MS scan (100–1600 m/z) with 60 000 resolution for parent ions followed by data dependent MS/MS for fragmentation of the seven most intense ions with collision-induced dissociation method.

### MS/MS protein identification

The LC-MS/MS data were submitted to a local mascot server for MS/MS protein identification via Proteome Discoverer (version 1.3, Thermo Fisher Scientific) against *Homo sapiens* (human) protein sequences contained within the Swissprot database. Typical parameters used in the Mascot MS/MS ion search were: trypsin digest with maximum of two miscleavages, cysteine carbamidomethylation, methionine oxidation, a maximum of 10 p.p.m. MS error tolerance and a maximum of 0.8 Da MS/MS error tolerance. A decoy database was built and searched. Filter settings that determine false discovery rates (FDR) are used to distribute the confidence indicators for the peptide matches. Peptide matches that pass the filter associated with the strict FDR (with target setting of 0.01) are assigned as high confidence. For MS/MS ion search, proteins with two or more high confidence peptides were considered unambiguous identifications without manual inspection. Proteins identified with one high confidence peptide were manually inspected and confirmed.

### Validation of isoform expression

#### Real-time quantitative reverse transcriptase-PCR analysis

Real-time PCR was performed in a total volume of 20 μl including 2 μl of cDNA, primers (0.2 mM each) and 10 μl of SYBR Green mix (Roche, Mannheim, Germany) using primer sets (Supplementary Table S6). Reactions were run on a Biorad CFX 96 machine using the following cycling parameters (95 °C for 5 min, 35 cycles of 10 s at 95 °C, 20 s at 60 °C and 15 s at 72 °C; melting curve: 10 s at 95 °C, 60 s at 60 °C). For quality-control purposes, melting curves for all samples were acquired. Transcripts levels were normalized to *Actb* and fold change was quantified using the standard curve method. The relative expression of each gene calculated as a fold difference between treated and untreated samples. Experiments were conducted in triplicate with at least three biological replicates.

#### Semi-quantitative PCR to measure isoform expression

mRNA expression analyses were performed as described [83]. In brief, total RNAs were isolated (QIAGEN RNeasy) (Qiagen, Vinlo, the Netherlands) from different experimental cells and digested with DNase1 prior to reverse-transcription reaction with qScript cDNA Supermix (Quanta Biosciences). Also total RNA from 0–12 h embryos from flies were isolated. The resultant cDNAs were used in semi-quantitative PCR with the indicated primer sets (Supplementary Table S6) and gels were stained with GelStar (Lonza). PCR cycling parameters were 95 °C for 5 min, 23 cycles of 10 s at 95 °C, 20 s at 55 °C (depending on the annealing temperature of the primers), and 15 s at 72 °C; 72 °C for 5 min and 4 °C forever. PCR products were resolved on 2% agarose gels and stained with GelStar Nucleic Acid Gel Stain. Splice isoforms were confirmed by cloning the products from PCR analyses using Topo blunt end cloning Kit (Invitrogen) according to manufacturer’s protocol and sequenced using the sanger method. At last, some of the detected transcripts are not annotated and were confirmed by sanger sequencing. Products from neighboring constitutive exons of the same genes were used to standardize for total transcription.

### *In silico* analysis

The following splice site prediction programs were used to predict the effect of variants on the efficiency of splicing: GeneSplicer (http://www.cbcb.umd.edu/software/GeneSplicer); Splice Site Prediction by Neural Network (http://www.fruitfly.org/seq_tools/splice.html); NNSPLICE 0.9 version (http://www.fruitfly.org/seq_tools/splice.html); and Drosophila Melanogaster Exon Database (http://proline.bic.nus.edu.sg/dedb/cgi-bin/viewer.py?id=13068). *I* *n silico* PCR program, http://genome.ucsc.edu/index.html?org=Hu​man&db=hg18&hgsid=142437216 was used to first obtain the predicted amplicon sizes before experimental PCR reactions were performed.

### Western blots

Western blots were performed per standard protocol and input dilutions were used as a quantitative indication of signal linearity. In brief, protein samples were resuspended in a reduced sample buffer 9 conatining SDS), and then electrophoresed on a Tris-glycine gel with Tris running buffer; blotted to PVDF membrane; and sequentially probed with primary antibodies against various proteins. Western blot-based detection was performed using alkaline phosphatase-coupled secondary antibodies with Vistra ECF for visualization, and images were obtained using a Typhoon 9400. ImageQuant 5.2 software was used to quantity of proteins in arbitrary units and relative protein concentrations were standardized to β-actin levels.

### Accession numbers

Data analyzed have been deposited in GEO with accession numbers GSE56120 and GSE56073.
