# Supplementary material for: Involvement of PARP1 in the regulation of alternative splicing
Source: Cell Discov. 2016 Feb 16;2:15046–. doi: 10.1038/celldisc.2015.46 (PMC4860959; doi:10.1038/celldisc.2015.46)
Supplement: Supplementary Figure S1 [file celldisc201546-s1.pdf]

Supplementary Figure 1

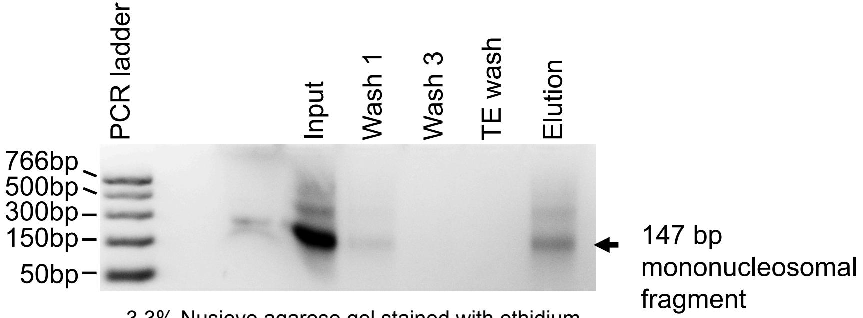

3.3% Nusieve agarose gel stained with ethidium bromide

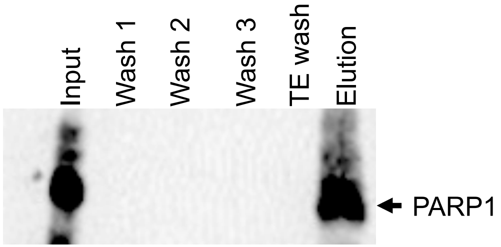

Western blot with anti-PARP1 antibody

Supplementary Figure S1: ChIP assay. Nuclei from S2 cells were fixed with 1% formaldehyde and chromatin digested with MNase. Chromatin was subjected to ChIP using anti-PARP1 antibody. A) Resultant DNA fragments from input, flow through, several wash steps and elution steps were purified and analyzed on 3.3% Nusieve™ agarose gel electrophoresis. DNA mononucleosomal fragment (arrow) from elution step was excised and subjected to ABI SoLiD™ sequencing. No mononucleosomal DNA was recovered from the elution in the IgG control experiment. B) Proteins from the ChIP experiments were subjected to western blot analyses and probed with PARP1 antibody.
