# Supplementary material for: Involvement of PARP1 in the regulation of alternative splicing
Source: Cell Discov. 2016 Feb 16;2:15046–. doi: 10.1038/celldisc.2015.46 (PMC4860959; doi:10.1038/celldisc.2015.46)
Supplement: Supplementary Figure S2 [file celldisc201546-s2.pdf]

Supplementary Figure S2

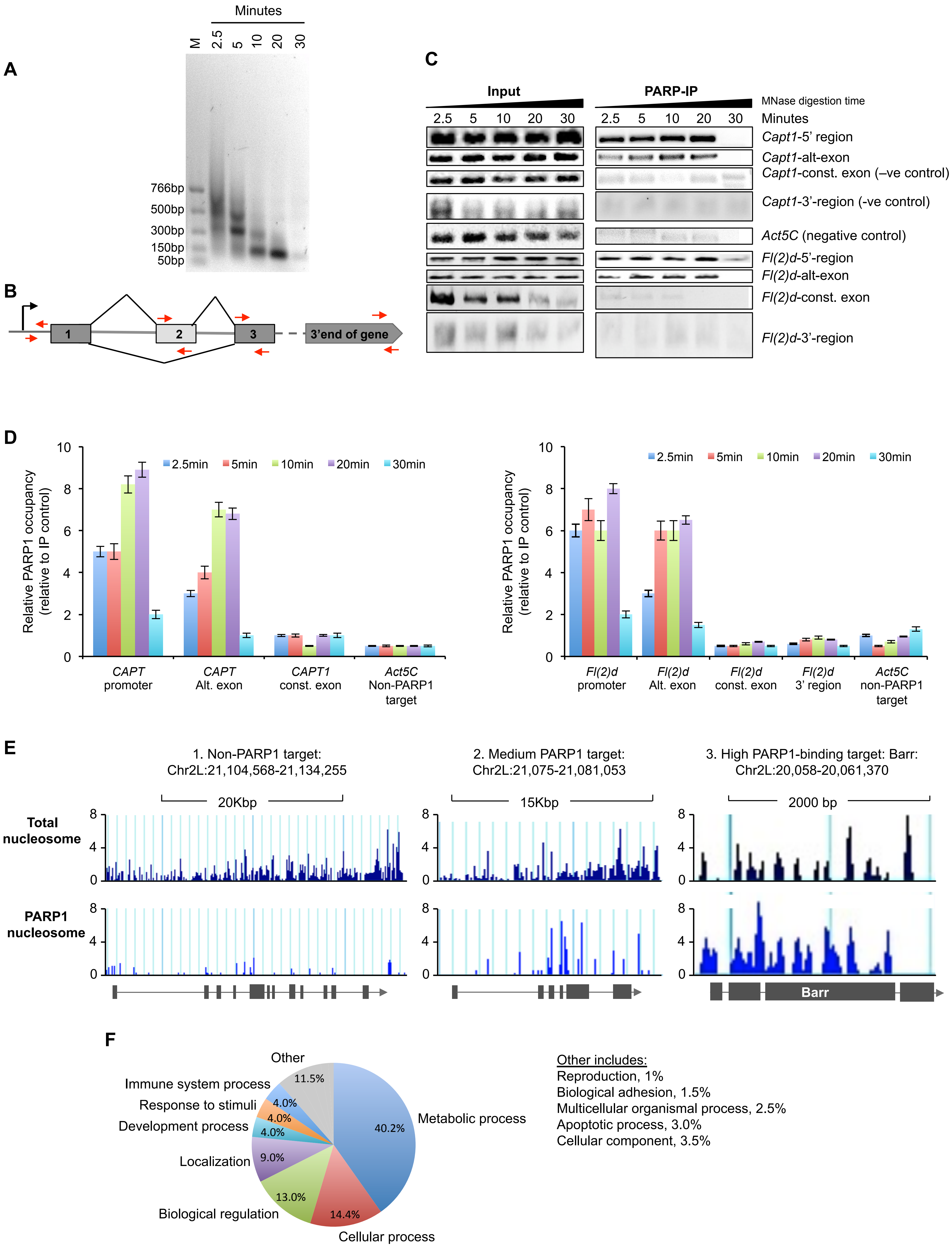

Supplementary Figure S2: Validation of PARP1 binding sites: A) Chromatin was digested with a titrated MNase time point of digestion. Digested chromatin was used on ChIP to determine the occupancy of PARP at selected genomic regions. B) An example of a gene structure showing the location of primers (red arrows) targeting specific genomic regions; From 5' - 3' – first primer set = first 5' nucleosome immediately following TSS; second primer set = targeting alternative exon; third primer set = targeting constitutive exon; fourth exon = 3' end of gene. C) Agarose gel images from ChIP at various MNase time points and specific primers. Negative controls include constitutive (const.) exons of Capt1 and Fl (2) d genes, non-PARP1 bound gene Act5c, 3'-region of both Capt1 and Fl (2) d depleted of nucleosomes and thus not pulled-down by PARP1 antibody). D) Real-time measurements of PARP1 occupancy at the various genomic regions of two representative genes and controls. Error bars are mean ± SD from two independent experiments. E) Snap-shots of PARP1 nucleosome and total nucleosome binding at three genomic regions 1. Low PARP1 binding gene 2. Medium PARP1 binding gene 3. High PARP1 binding gene. F) Functional pathway of PARP1-target genes from PARP1-nuc-ChIP-seq (Fig. 2A).
