# Supplementary material for: Involvement of PARP1 in the regulation of alternative splicing
Source: Cell Discov. 2016 Feb 16;2:15046–. doi: 10.1038/celldisc.2015.46 (PMC4860959; doi:10.1038/celldisc.2015.46)
Supplement: Supplementary Figure S3 [file celldisc201546-s3.pdf]

Supplementary Figure S3

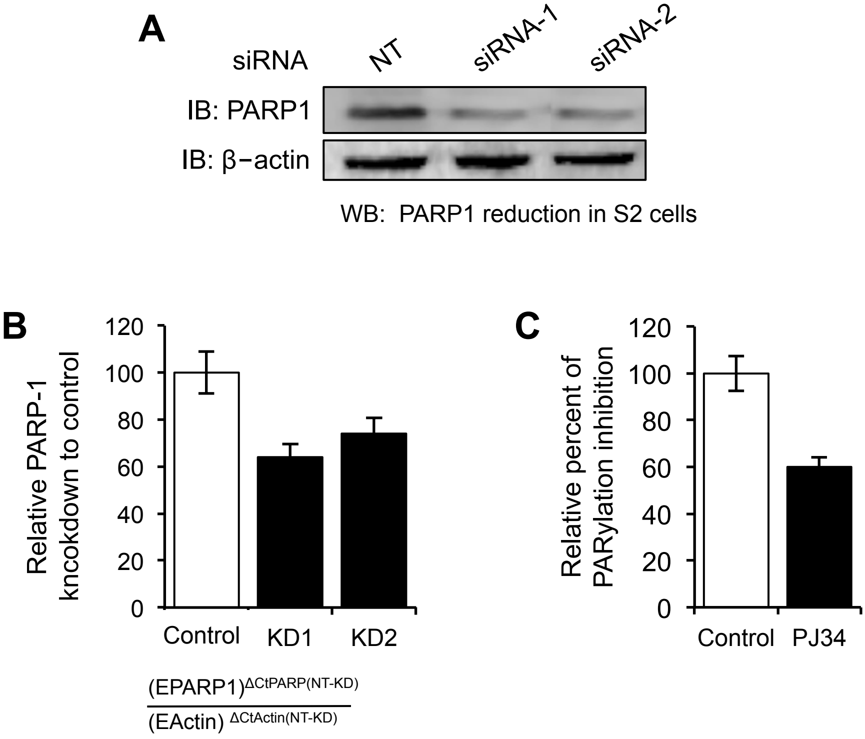

Supplementary Figure S3: Knockdown of PARP1 and inhibition of PARylation. Cells were transfected with non-targeting LacZ siRNA (NT) and PARP1 siRNA and the expression of PARP1 was measured either by: **A)** Western blot analyses for protein levels. Equal amount of lysates by total protein content was loaded on an SDS PAGE gel. Proteins were probed for PARP1 presence with anti-PARP1 antibody. Concentrations of PARP1 were normalized relative to β-actin level. or **B)** Quantitative real-time PCR was used to measure PARP1 mRNA levels. Total RNA was harvested from control and treated cells, and converted to cDNA (SuperScript III reverse transcriptase from Invitrogen). qRT-PCR was performed with cDNA from control and treated cells for PARP1 siRNA. Expression results were normalized to β-actin mRNA levels. **C)** Cells were treated with PARylation inhibitor and levels of PARylation measured using PARP Pharmocodynamic assay kit from Trevigen®. Error bars are mean ± SD from three independent experiments.
