# Supplementary material for: Involvement of PARP1 in the regulation of alternative splicing
Source: Cell Discov. 2016 Feb 16;2:15046–. doi: 10.1038/celldisc.2015.46 (PMC4860959; doi:10.1038/celldisc.2015.46)
Supplement: Supplementary Figure S4 [file celldisc201546-s4.pdf]

Supplementary Figure S4

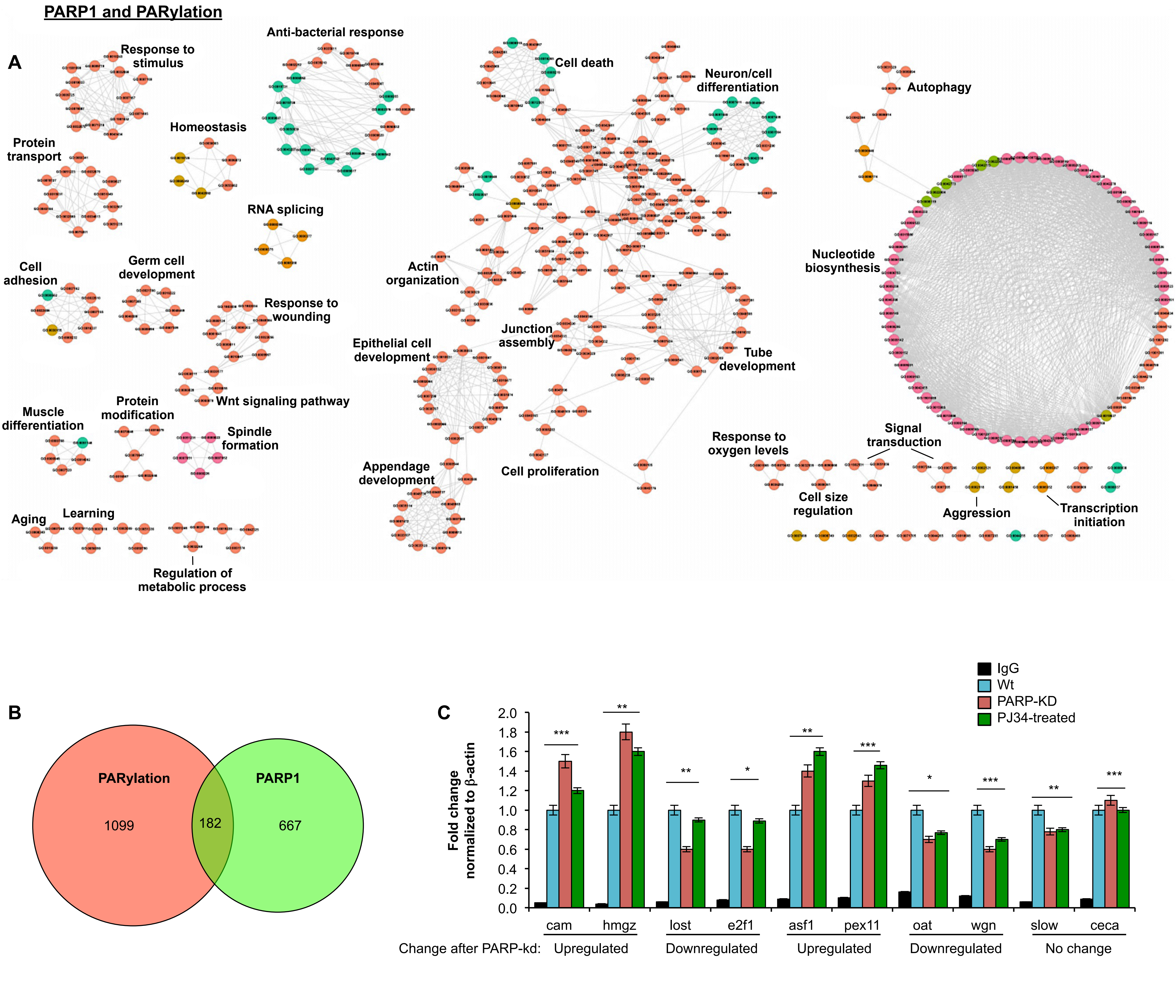

Supplementary Figure S4: A) Visualization using categoryCompare of the functional profiles of DEGs mediated by PARP1 and PARylation. Color code represents the functions of the DEGs: Pink: PARP1 upregulated genes; red-orange: PARP1 downregulated genes; Orange: PARylation upregulated genes; Gold: PARylation downregulated genes; Mint: PARP1 and PARylation commonly down-regulated genes; Pea Green: PARP1 and PARylation upregulated genes. B) Venn diagram illustrating the overlaps of the genes with expression levels that were uniquely or concordantly regulated by PARP1 and/or PARylation (**t**-test, **p** < 0.05). C) Validation of changes in the expression of genes after PARP1 kncokdown and PARylation inhibiton. Shown are results from three independent experiments and error bars are the ± SD (p < 0.05 from student t-test).
