# Supplementary material for: Involvement of PARP1 in the regulation of alternative splicing
Source: Cell Discov. 2016 Feb 16;2:15046–. doi: 10.1038/celldisc.2015.46 (PMC4860959; doi:10.1038/celldisc.2015.46)
Supplement: Supplementary Figure S5 [file celldisc201546-s5.pdf]

Supplementary Figure S5

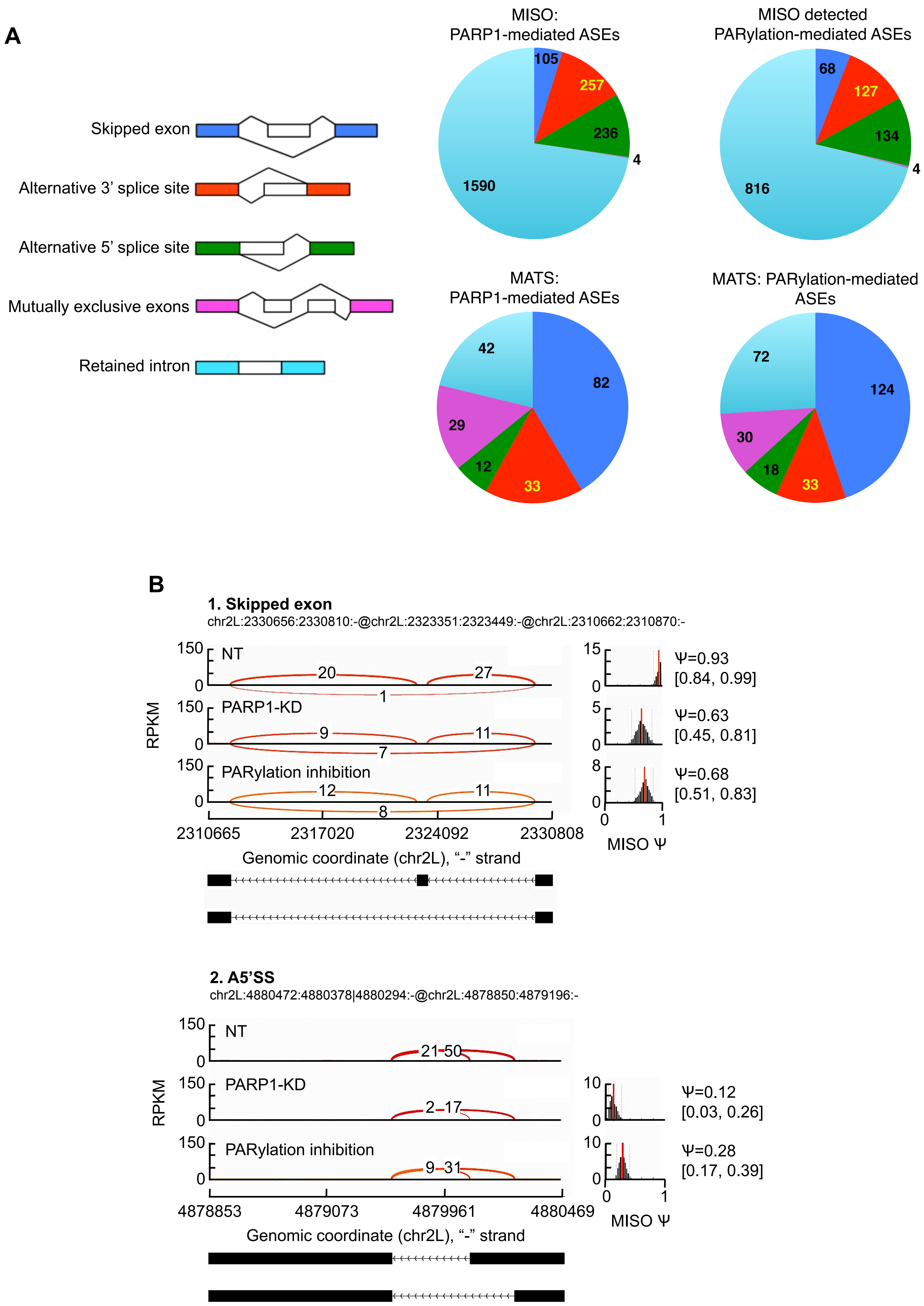

Supplementary Figure S5: Splicing events observed after PARP1 Knockdown and PARylation inhibition. A) Venn diagram showing the different types of ASEs that are detected either by MATS or MISO detected by both methods. B) Sashimi plots showing examples of ASEs mediated by PARP1 and PARylation. RNA-seq read densities supporting ASEs and the estimated confidence levels are shown in the figure.
