# Supplementary material for: Involvement of PARP1 in the regulation of alternative splicing
Source: Cell Discov. 2016 Feb 16;2:15046–. doi: 10.1038/celldisc.2015.46 (PMC4860959; doi:10.1038/celldisc.2015.46)
Supplement: Supplementary Figure S6 [file celldisc201546-s6.pdf]

Supplementary Figure S6

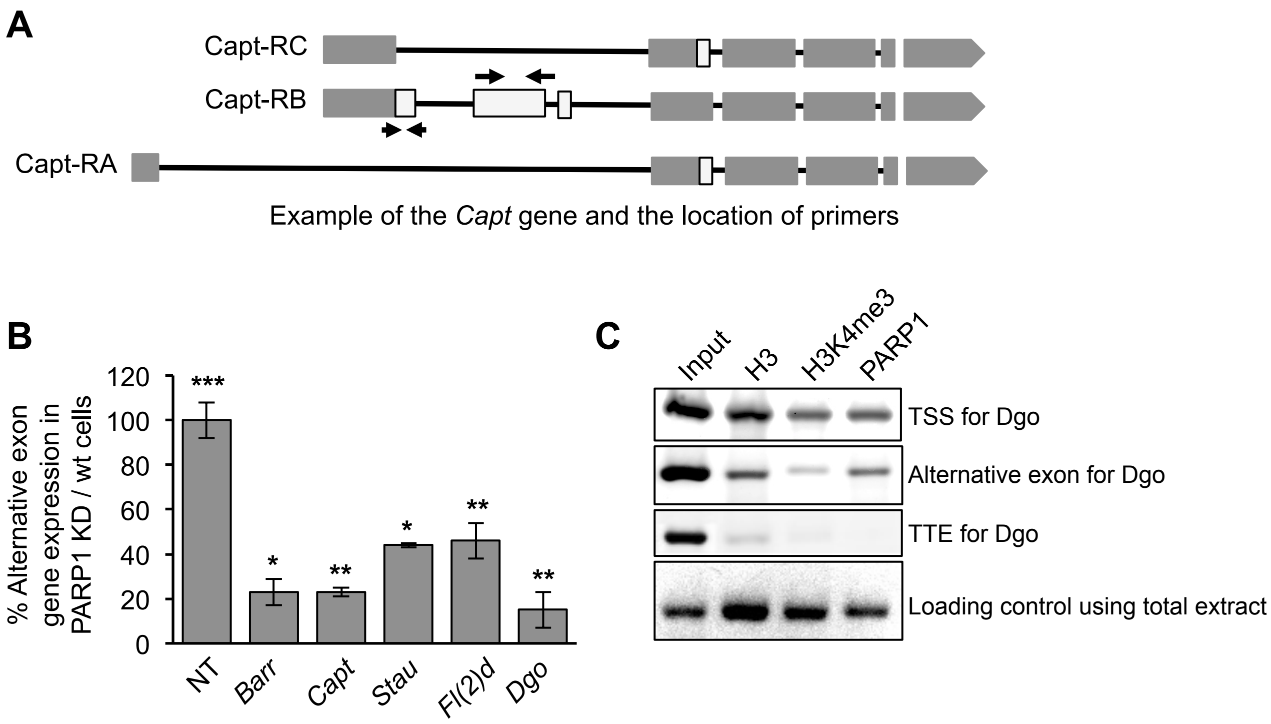

Example of the *Capt* gene and the location of primers

Supplementary Figure S6: Validation of the RNA-seq data on alternative splicing patterns using Quantitative real-time PCR (qRT-PCR). A) *Capt*, an example of a gene in which PARP1 binds to the internal exon/intron boundaries. Illustration of the different splice variants and the design of primers used to detect the different splice isoforms. **B)** Real-time measurement of the gene expression from the various alternative exons of the various genes tested in Figs. 5. Error bars are mean ± SD from three independent experiments. *, **, *** Indicate significance of P<0.05, p<0.005 and p<0.0005 respectively by Student's t-test in gene expression in control cells (using non-targeting siRNA) vs. PARP1 siRNA or PJ34 treated cells. **C)** Some PARP1-target genes do not have H3K4me3 at the exon-intron boundaries. Shown is a representative gel measuring the occupancy of H3K4me3 at PARP1 targets – TSS and alternative exon of *Dgo*; and at non-PARP1 target regions (TTE- transcription termination end and constitutive exon of *Dgo*.
