# Supplementary material for: Involvement of PARP1 in the regulation of alternative splicing
Source: Cell Discov. 2016 Feb 16;2:15046–. doi: 10.1038/celldisc.2015.46 (PMC4860959; doi:10.1038/celldisc.2015.46)
Supplement: Supplementary Figure S7 [file celldisc201546-s7.pdf]

Supplementary Figure S7

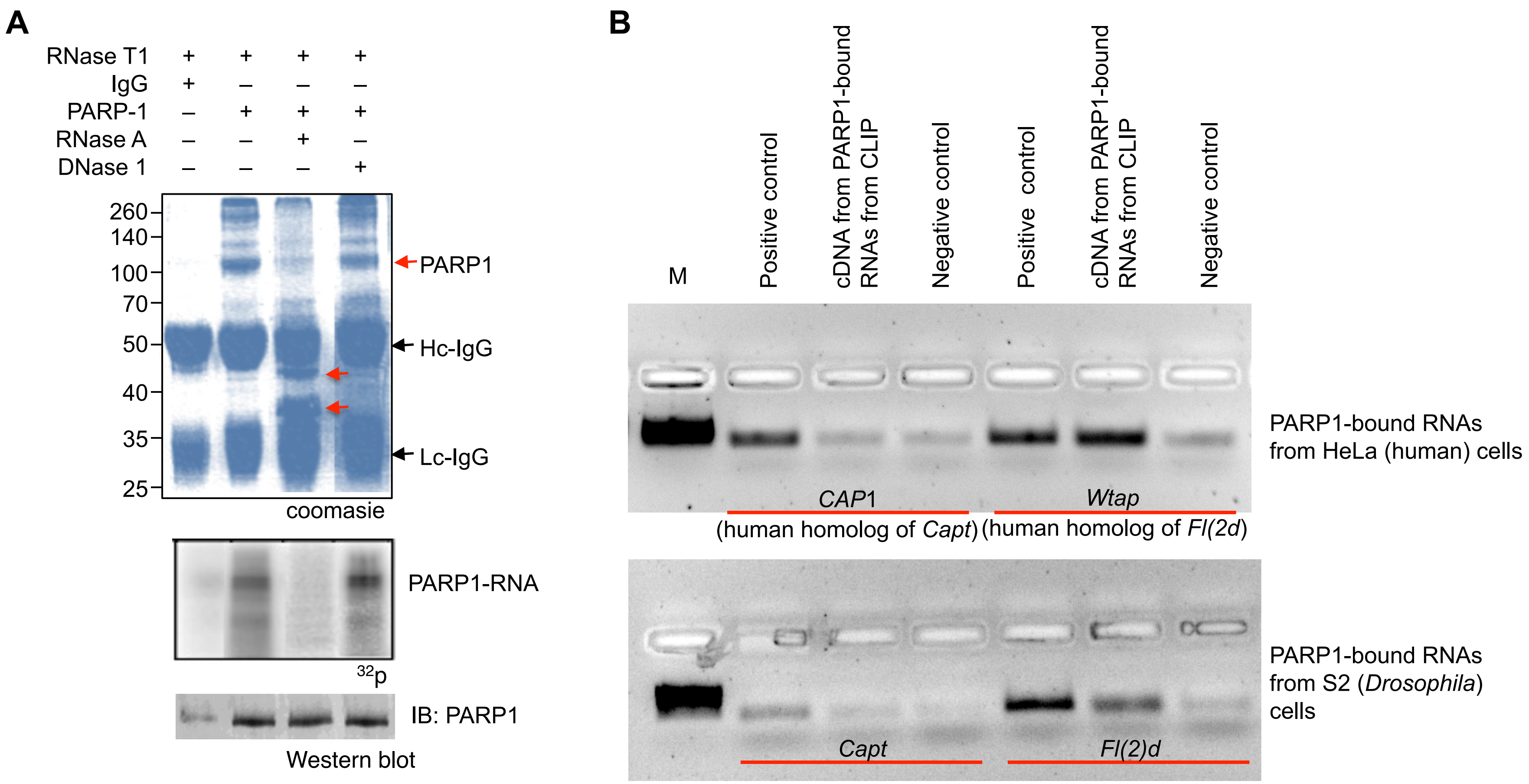

Supplemental Figure S7: PARP1 binds nascent mRNA in *Drosophila* S2 cells and human HeLa cells. **A)** PAR-CLIP experiments were carried out in Drosophila S2 and Human HeLa cells using IgG and PARP1 antibody. Samples resulting from PARP1 antibody were split into three aliquots i) no further treatment; ii) further stringent DNase1 treatment; iii) further stringent RNaseA. Knockdown of PARP1 as well as stringent RNase treatment eliminated the PARP1-RNA band. **B**) PARP1 bound RNA (nucleic acids) from both human HeLa and Drosophila S2 cells were purified, reversed transcribed to cDNA and subjected to RT-PCR. Specificity of PARP1 binding to the mRNA of its target genes was validated using RT-PCR and primers specific for some exons of PARP1 target genes. As control, RNA before reverse transcription was used to control for background effects due to contaminating DNA. Total RNA from both cell types was reverse transcribed and used as positive control.
