# Supplementary material for: Involvement of PARP1 in the regulation of alternative splicing
Source: Cell Discov. 2016 Feb 16;2:15046–. doi: 10.1038/celldisc.2015.46 (PMC4860959; doi:10.1038/celldisc.2015.46)
Supplement: Supplementary Figure S8 [file celldisc201546-s8.pdf]

Supplementary Figure S8

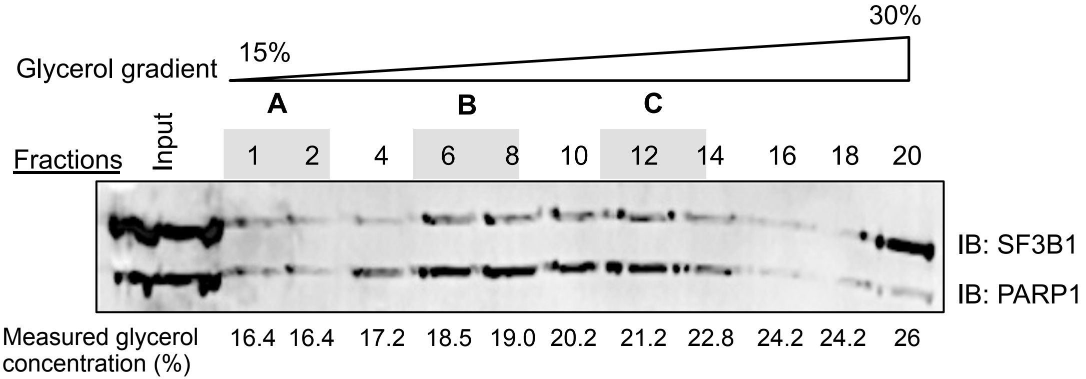

Supplementary Figure S8: Co-sedimentation of PARP1 and SF3B1. Fractionation of HeLa nuclear extracts on a 10 - 30% glycerol gradient, shows that PARP1 co-sediments with SF3B1. Glycerol concentrations in each aliquot as measured by refractory indices are shown. Protein markers: A= 5.4S (conalbumin), B= 6-8: aldolase and C= 11.5S.
