# Supplementary material for: Involvement of PARP1 in the regulation of alternative splicing
Source: Cell Discov. 2016 Feb 16;2:15046–. doi: 10.1038/celldisc.2015.46 (PMC4860959; doi:10.1038/celldisc.2015.46)
Supplement: Supplementary Table S1 [file celldisc201546-s9.pdf]

Additional File 9

Table S1: Genes bound at their internal exon-intron boundaries by PARP-1

CG7718	CG30291	CG9279	Nmdmc	Ssl1
Inos	Hexo1	CG7173	4EHP	CycB
CstF-50	CG5746	CG9890	CG11141	ntc
pll	CG34386	CG12983	Scm	CG15386
RpL34a	Cyp312a1	CG6761	Snap29	CG1142
CG1620	Rrp6	syd	exu	CG10249
dco	Cand1	stau	Aprt	Hr78
CG32428	Exo84	CG30499	dream	CG32196
Sply	CG9467	sel	CG6923	Acp63F
CG3214	CG8485	CG10904	CG31636	CG10151
CG13771	CG5384	Acph-1	CycG	CG42815
pita	CG7987	CG13044	CG7601	CG7180
CG34135	CG5773	dpa	XNP	spen
CG3338	Pl31	KLHL18	CG11247	CG2915
CG8441	mRpS18B	CG6424	CG8207	Cys
fl(2)d	CG10625	lwr	Ravus	barr
janA	CG32846	pip	CG4050	DnaJ-H
CG4038	CG9306	Prosbeta2R2	CG3955	Trl
CG5195	CG4538	mira	p24-2	Prosbeta7
SelR	iPLA2-VIA	pnt	CG5506	Hrs
Atac3	can	CG6767	CG8841	CG4360
CG3397	CG9897	fdl	Vha16-1	Tes
CG9422	kni	egr	CG12736	CG13397
CG12512	wfs1	La	cdm	D1
CG9799	Drl-2	CG9760	dgo	spict
CG32459	CG8850	CG3501	az2	CG8768
Gfat2	RpL6	CG6791	R	lov
Pdk1	CG42708	mfr	CG3645	Cul-5
CG5835	CG5554	Bre1	asl	CG5968
CG31122	eIF-4E	mRpL23	Keap1	Prm
achi	Cp190	RpS13	CG8230	wtrw
CG1317	CG13157	CG13311	calypso	CG32364
CG30460	SH3PX1	CG18268	ltd	shark
