# Supplementary material for: Involvement of PARP1 in the regulation of alternative splicing
Source: Cell Discov. 2016 Feb 16;2:15046–. doi: 10.1038/celldisc.2015.46 (PMC4860959; doi:10.1038/celldisc.2015.46)
Supplement: Supplementary Table S2 [file celldisc201546-s10.pdf]

Table S2: Differential expressed genes modulated by PARP-1 KD and PARylation inhibition

Total of 20,706 Transcriptional start sites

PARylation dataset: 1,281 Differentially expressed genes (p-value <= 0.05) (974 up-regulated; 307 down-regulated)

PARP Knockout dataset: 849 Differentially expressed genes (p-value <= 0.05) (210 up-regulated; 639 down-regulated)

Table S2 - A: Genes up-regulated in PARylation dataset

Aats-asn	CG12643	CG1648	CG31917	CG41257	CG6180	CR44197
CG1674	CG31997	CG16713	CG31957	CG4186	CG6272	Yeti
CG12728	CG12721	CG6196	CG31955	CG41378	CG16712	ZetaCOP
AlkB	AGO3	CG32039	CG12825	CG16753	CG1265	ox
Anp	Ada1-2	CG42394	CG42375	CG6357	CG12824	laccase2
Alg-2	Acyp2	CG16782	CG32038	CG42363	CG6310	p120ctn
Ak6	Act79B	CG12859	CG16758	CG32000	CG42336	Mpp6
Aph-4	CG32091	CG4250	Apf	CG1707	CG6463	mRpL10
CG6685	CG17167	CG3209	CG6583	CG12948	CG42394	mRpL14
CG42502	CG12975	CG17121	CG42495	CG32068	CG16817	mRpL17
aph-1	CG6610	CG12970	CG32085	CG12868	CG6424	mRpL20
CG17350	CG13283	CG17323	CG42566	CG6833	CG6812	mRpL21
CG13315	Arpc3B	CG17294	CG42553	CG32147	CG6770	mRpL23
asf1	CG13123	CG32163	CG42550	CG32115	CG17282	mRpL24
Ast	CG17189	CG13014	CG13117	Arf84F	Arf102F	mRpL32
CG42792	Atox1	CG13364	CG7024	CG1738	CG6836	mRpL33
CG32267	Atf6	CG17385	CG7006	CG13339	CG42581	mRpL34
CG17490	CG17454	CG32243	CG42740	CG6999	CG32202	mRpL36
CG13510	CG13365	CG42790	CG32230	CG4269	CG32196	mRpL39
CG7320	CG32313	B4	aub	CG17471	CG42807	mRpL40
CG42821	CG17490	CG7194	CG13506	CG32276	CG7137	mRpL42
babos	CG13551	CG17508	CG32364	CG43088	CG7381	mRpL46
baf	CG13559	CG17556	CG32440	CG43107	CG7408	mRpL49
Best4	CG13599	CG17597	CG32500	CG43124	CG7484	mRpL54
Bet5	CG13603	CG17625	CG32582	CG43156	CG7519	mRpL55
betaNACtes3	CG13663	CG17691	CG32590	CG43165	CG7603	mRpS14
betaNACtes6	CG13689	CG17698	CG32625	CG43210	CG7630	mRpS17
Bgb	CG13733	CG17715	CG32647	CG43246	CG7646,	mRpS18A
BI-1	CG13773	CG17737	CG32706	CG4325	CG7668	mRpS18C
bib	CG13794	CG17760	CG32708	CG43266	CG7713	mRpS21
Bin1	CG13822	CG17776	CG32718	CG43273	CG7770	Drs
blos3	CG13876	CG17777	CG32736	CG43291	CG7772	Drsl5
blos4	CG13890	CG17834	CG32756	CG43295	CG7787	dyn-p25
boca	CG13896	CG17883	CG32822	CG43336	CG7824	e(y)2
c12.1	CG13993	CG17977	CG32832	CG43446	CG7949	eater
Cals	CG14022	CG18081	CG32856	CG4362	CG8089	eIF-5A
CaMKII	CG14036	CG18190	CG32982	CG43647	CG8145	Eip71CD
Caps	CG14057	CG18213	CG33051	CG43886	CG8152	elm
Caps	CG14103	CG1827	CG33107	CG4390	CG8204	Ephrin
cbc	CG14105	CG18316	CG33129	CG43921	CG8213	epsilonCOP
CCS	CG14118	CG18507	CG33136	CG44006	CG8321	fan
cdc2	CG14130	CG18549	CG33169	CG44102	CG8358	Fibp
cerv	CG14182	CG18600	CG33217	CG4592	CG8369	Fis1
CG10157	CG14210	CG18619	CG33225	CG4593	CG8436	FKBP59
CG10195	CG14270	CG18622	CG33275	CG4598	CG8455	Flo-1
CG10205	CG14286	CG18731	CG33460	CG4646	CG8461	fs(1)Ya
CG10222	CG14291	CG18765	CG33461	CG4692	CG8498	fsd

CG10283	CG14309	CG18810	CG33462	CG4793	CG8507	FucTB
CG10320	CG14407	CG1969	CG33469	CG4858	CG8586	FucTC
CG10375	CG14410	CG2046	CG33494	CG4935	CG8620	fy
CG10418	CG14434	CG2177	CG33552	CG4949	CG8675	Gas41
CG10465	CG14464	CG2200	CG33774	CG4953	CG8680	Gbp
CG10479	CG14483	CG2219	CG33926	CG4957	CG8768	gdl,gdl-ORF39
CG10495	CG14545	CG2316	CG33977	CG4970	CG9034	Gos28
CG10550	CG1458	CG2371	CG34031	CG5013	CG9117	Oscillin
CG10592	CG14615	CG2453	CG34039	CG5039	CG9170	Gpi1
CG10635	CG14671	CG2608	CG34054	CG5045	CG9231	Gprk1
CG10764	CG14721	CG2709	CG34115	CG5056	CG9267	GstD2
CG10802	CG14795	CG2789	CG34117	CG5073	CG9344	GstD5
CG1105	CG14812	CG2974	CG34132	CG5131	CG9350	G44206
CG11077	CG14817	CG30001	CG34148	CG5149	CG9393	veli
CG11151	CG14818	CG30016	CG34159	CG5150	CG9463	Vha14-1
CG11170	CG14860	CG30022	CG34163	CG5285	CG9505	VhaM9.7-b
snoRNA:Psi 28S-2149	snoRNA:Psi 18s-1377d	snoRNA:Psi 18s-1389a	snoRNA:Psi 18s-1397	snoRNA:Psi 28s-1837c	snoRNA:Psi 525e	snoRNA:Psi 18S-525g
CG11279	CG14903	CG30088	CG34195	CG5343	CG9586	CG30046
CG1129	CG14977	CG30090	CG34213	CG5375	CG9616	CG34191
CG11319	CG14985	CG30096	CG34228	CG5382	CG9624	CG5323
CG11329	CG14985	CG30109	CG34242	CG5385	CG9631	MFS17
CG11395	CG15019	CG30187	CG34250	CG5412	CG9766	mgl
CG1142	CG15020	CG30195	CG34293	CG5463	CG9782	mip40
CG11455	CG1503	CG30269	CG34305	CG5515	CG9796	Mis12
CG11577	CG15065	CG30273	CG3436	CG5535	CG9804	Mitf
CG11668	CG15107	CG30373	CG34371	CG5626	CG9815	Mob3
CG11686	CG15168	CG30383	CG34398	CG5630	CG9836	MP1
CG11710	CG15220	CG30392	CG34436	CG5676	CG9837	Npc2b
CG11755,	CG15317	CG3040	CG34437		CG9989	nrv3
CHIP	CG5697	CG5708	CG34454	CG30403	CG1532	nuf
CG11781	CG5846	CoVa	CG5861	CR31451	CG5869	Nup43
CG11802	CG15323	CG30424	CG3457	CG5727	Chrac-16	Nxt1
CG11839	CG15345	CG31111	CG3476	CG5731	CkIIalpha	Obp18a
CG11920	CG15347	CG3117	CG3621	CG5783	CkIIalpha-i3	Jafrac2
CG11985	CG15362	CG31174	CG3678	CG5844	cni	Kmn1
CG11999	CG15386	CG31221	CG3740	cold	CG5885	l(1)10Bb
CG12112	CG15440	CG31229	CG3812	CORL	CR34335	Nf-YB
CG31324	CG31360	CG12206	Gie	mago	CR44101	Hsp70Bb
CG3884	CG31370	CG12224	Nca	Dbp80	FK506-bp2	MED10
CG3887	CG31460	CG12253	obst-A	FK506-bp2	mRpL41	Crk
CG3939	CG31469	CG12279	Ogg1	Vps25	Oxp	CSN8
CG40002	CG31717	CG12304	hoe1	vps2	daw	SmG
CG40045	CG15456	mRpS26	HP1b	CR43995	Lsm10	sn
CG5969	CG15514	l(3)72Dh	Hsc70-2	Lrr47	CR43967	snk
CG5941	CG15535	mRpS28	Hsp26	Lip4	loj	snoRNA:
CR40469	CG15536	mRpS33	Nnf1a	CR43957	lin-52	gp210-a
CR42861	CG15715	mRpS35	O-fut1	CR43857	CR43907	snoRNA:

CG40178	CG15766	mRpS5	olf413	CR43785	lectin-28C	lola-c
CG5973	CG15818	mRpS25	NHP2	MED4	lectin-24Db	snoRNA:
CG40127	CG12129	CG8229	l(3)87Df	MED26	Mes4	Or-aca1
CG31715	CG12171	CG33199,	zye	MED22	MED7	Dcp1
CR43417	CG6142	CG31778	CG12520	CR43157	CR43148	Det
CR43497	CG40228	CG15908	CG12567	CG40191	CG12379	Dgp-1
CG31807	CG40439	CG15922	CR43264	CG31777	CG1599	dgt2
CG6171	CG31800	CG12535	CG6005	CG15881	CG5984	Dhfr
CG12594	CG1607	CG3184	CG41128	Rap2l	CG41099	Dip2
Rpp30	CG14894	CG9510	Rcp	spn-D	CR43769	dj-1beta
rpr	CG11263	rl	Rgk1	spt4	Spn88Eb	HSPC300
snoRNA: Psi 18S-996 RpS15Ab	snoRNA:Psi 28S-1135e Sra	snoRNA:Psi 2442b P32	snoRNA:Psi 28S-2444 M2	snoRNA:Psi 28S-2562 IM3	snoRNA:Psi 28S-2648 p38c	IsnoRNA:Psi 28S-3327c Sras
RpS19b	robl	roX2	Roc2	Sr-CI	Pa1	Inr-a
RpS2	Pbgs	Srp9	Rpb10	Srp19	Parp	Ir41a
RpS21	Ir92a	RpS30	Rpb11	srpk79D	Pcd	Obp56h
snoRNA:Psi 28S-1175	snoRNA:Psi 1135f	snoRNA:Psi 28S-3342	snoRNA:Psi 28S-1175	snoRNA:Psi 28S-3385a	SnoRNA:Psi 28S-612	snoRNA:Psi 1175c
Rpt6R	Ssb-c31a		Rpb12	pen-2	Pex11	kn
SnoRNA:Psi 28C-1192c	snoRNA:Psi 1175b	snRNA:U2 34ABa	tko	SnoRNA:Psi 28S-1232	SnoRNA:Psi 1192a	SnoRNA:Psi 28S-1837a
rumi	Rrp47	Pur-alpha	Rpb8	Stlk	Pgk	nerfin-2
rush	Rpb7	r2d2	Rpl12	Su(P)	Pho	MED11
SamDC	ssp	Rab18	RpII18	sun	pim	Max
Sclp	Tim9a	Rab19	RpII33	Taf10b	Taf11	crok
Scp1	Pgam5	Rab30	RpL15	Taf13	RpL10	Tsp29Fa
SdhC	TotA	Sirt4	RpL21	Tango4	pinta	Tsp86D
sea	TotB	Sirt6	RpL23	Tbh	png	ubl
Sec61gamma	TotM	slam	RpL24-like	Tep1	pnn	Ucrh
SelG	TpnC4	SmD1	RpL29	TfIIA-S	poly	Uev1A
sept, 4	TpnC41C	slam	RpL37A	TfIIA-S-2	pot	uex
SnoRNA:Or aca3	SnoRNA: Prp8-a	SnoRNA: Psi 1837a	snoRNA:Psi 1377a	snRNP-U1-C	SnoRNA: Psi 18S-110	Uhg1
shg	Trs23	slam	RpL39	Tif-IA	Pros28.1	Uhg2
CR43958,	trsn	MED21	cta	RpL41	Prosalpha5	Uhg3
Thd1	Trx-2	slam	Socs36E	RpLP0-like	Tim17b	Uhg4
GstD6	LSm7	l(3)72Do	Sod	CG42846	Tim8	mRpS7
GstD7	Cyp6a23	slam	Spase22-23	TfIIB	Prosbeta1	msd1
GstE7	Cyt-b5	CG42568	Spf45	Dlc90F	Prp18	Msr-110
GstE8	Cyp6a14	slam	Uhg5	CG7339	vps28	mtacp1
GstT1	MED28	SmF	Uhg8	CG17048	wibg	mthl15
GstZ1	Cyp4ac2	slam	UK114	CG32069	yellow-f	Mul1
GXIVsPLA2	SmE	DnaJ-60	CG10576-a	CG42490	yellow-f2	myo
Haspin	Sgt1	trbl	upd2	CG6480	HP4	MYPT-75D

hebe	RpL38		upd3	CG12883	Hsp23	nahoda
His4r						NC2beta
HmgZ	CR44127	ORMDL	CG42868	mex1	org-1	Ndg
NijA	CR44197	CG32350	bab2	CR44030	maf-S	nemy
NHP2	CG8043	CG17493	CG13516	Ama	Maf1	Nipped-B
l(2)k10201	SmD2	CR44134	usnp	Hsp67Bc	lt	l(2)k12914

Table S2 - B: Genes down-regulated in PARylation inhibition dataset

Ac76E	CG13097	CG32206	CG5794	CLIP-190	gsb	Myo31D F
Acon	CG13124	CG3226	CG5955	corn	gw	nAcRbet a-21C
Acsl	CG13252	CG3290	CG6045	CR31144	Hel89B	nAcRbet a-96A
Act42A	CG1340	CG3301	CG6361	cu	Hml	nes
AIF	CG13458	CG33116	CG6479	Cul-3	Hr51	Nhe2
Aldh-III	CG1358	CG3376	CG6656	CycE	Hrb87F	nimB2
Alk	CG14082	CG34198	CG6659	Cyp12a5	Hsc70-4	nimB3
alpha-Est7	CG14085	CG34212	CG7056	Cyp12c1	htl	Npc2g
alphaTub84D	CG14221	CG34227	CG7530	Cyp6a21	Indy	nudE
aop	CG14273	CG34232	CG7737	Cyp6d2	kek1	Oat
Arc1	CG14439	CG34330	CG7778	Cyp9c1	Klp3A	Orct2
arr	CG14440	CG34383	CG7794	dally	kst	out
asp	CG14442	CG34417	CG7839	debcl	l(2)k09022	Pask
AttA	CG14629	CG3505	CG7872	Dg	l(3)72Ab	Pax
AttB	CG14712	CG3509	CG8034	dmGlut	lea	Pfk
AttD	CG14741	CG3703	CG8051	DNApol-epsilon	LKR	pgant6
Axud1	CG15308	CG3847	CG8086	dock	LM408	PGRP-SB1
betaTub97EF	CG15365	CG3984	CG8108	dpr17	Lpin,kermit	PI4KIIIal pha
bgm	CG15366	CG40160	CG8157	DptB	lva	Pp2C1
bmm	CG15695	CG4096	CG8160	Drep-1	mAcR-60C	Ppcs
bnl	CG15740	CG42327	CG8552	Dro	Mat89Ba	pxb
caz	CG15784	CG42450	CG8939	Dscam	Mcm6	pyd3
Cdc6	CG16700	CG42613	CG8944	egh	Mdh2	qless
CG10178	CG16799	CG42715	CG8963	egr	Mec2	Ranbp9
CG10339	CG16857	CG42788	CG9086	ETHR	MED26	Rapgap1
CG10383	CG16868	CG4297	CG9171	Fas2	mew	RecQ4
CG10657	CG17181	CG43175	CG9270	fat-spondin	mfas	Reg-5
CG10702	CG17337	CG43236	CG9312	Fhos	MFS3	RhoGAP 93B
CG1091	CG17544	CG43444	CG9331	Fkbp13	Mgstl	Rip11
CG11897	CG18605	CG43462	CG9338	Fmo-2	Mlc2	robo
CG12116	CG2865	CG4500	CG9626	fog	mod(mdg4)	Rpl135
CG12121	CG30089	CG4679	CG9717	Fuca	mRpS30	Rpn1
CG12173	CG30118	CG4752	CG9733	GlcAT-P	Msh6	scaRNA: pUf68-a

CG12194	CG30431	CG4797	CG9915	glec	msk	sda
CG12263	CG30484	CG4825	chb	Glt	Mtk	sdk
CG12325	CG3091	CG4829	CHKov1	Glycogenin	MtnB	Ser
CG12499	CG31038	CG5001	chrb	GlyP	Muc4B	sick
CG13078	CG31098	CG5080	ClC-a	Gpdh	Muc68Ca	siz
CG13096	CG32165	CG5599	slow	SMSr	snoRNA:229	stj
snoRNA:Me28S-U1230	snoRNA:Psi18S-1377e	snoRNA: Psi18S-1347a	snoRNA: Psi18S-525f	snoRNA:Psi18S-920	snoRNA:Psi28S-1060	snoRNA: Psi28S-1135b
zormin	zfh1	yrt	Ykt6	wgn	wds	vfl
Ugt86Di	Ugt86Da	Ugt58Fa	Tsf1	trol	Traf4	Tina-1
timeout	Tig	Tep5	tara	Tab2	su(r)	Spn100A

Table S2 - C: Genes up-regulated in PARP-1-KD dataset

A16	CG13779	CG33462	CG7056	DIP1	mRpL27	RpL18A	Ama
alphaTub67C	CG13994	CG33509	CG7172	eca	mRpL52	RpL28	CG14309
snoRNA:Psi28S-1192a	snoRNA:Psi28S-1192c	snoRNA:Psi28S-1192d	snoRNA:Psi28S-2149	snoRNA:Psi28S-2444	mRpS21	RpL34a	CG3645
Aplip1	CG14340	CG3817	CG7342	Eip55E	Msr-110	RpL34b	CG7299
ATPsyn-Cf6	CG14543	CG3884	CG7580	Eip71CD	Mst36Fb	RpL35A	Ef1beta
ATPsyn-d	CG14561	CG4068	CG7712	ETHR	mtg	RpL36A	CCHa2r
awd	CG14715	CG42336	CG7794	galectin	Muc4B	Rpn12	CG1600
Best4	CG14795	CG43143	CG8147	Gbs-76A	nau	RpS12	CG4884
bib	CG14946	CG43795	CG8157	glob1	nemy	RpS19b	CG8397
boca	CG15308	CG4500	CG8213	Gs2	nerfin-2	RpS20	HmgZ
Cam	CG15634	CG4793	CG8331	Gyc-89Da	Nhe1	RpS24	oa2
snoRNA:Psi28S-2648	snoRNA:Psi28S-3186	snoRNA:Psi28S-3327b	snoRNA:Psi28S-3436a	snoRNA:Psi28S-3436b	snoRNA:Psi28S-612	scaRNA:46E3	snRNA:U2:34ABa
CG10265	CG1835	CG4980	CG8501	HP6	obst-E	sick	Spase25
CG10512	CG18557	CG5010	CG8860	ial	p130CAS	SmF	Spn31A
CG10592	CG18605	CG5321	CG8963	Ir7g	Pal2	SmG	Spn88Eb
snoRNA:Psi28S-1175a	snoRNA:Psi18S-1854c,	snoRNA:Psi18S-1854b	snoRNA:Psi18S-1820	snoRNA:Psi18S-1377e j	snoRNA:Psi18S-1377c	snoRNA:Psi18S-1347c	snoRNA:Psi18S-1377a
snoRNA:Psi28S-1135b	snoRNA:Psi28S-1060	snoRNA:Psi18S-920	CG9065	ksh	Pomp	Srp14	su(r)
CG11671	CG2812	CG5862	CG9119	l(2)35Di	ppk13	png	sun
CG11672	CG30015	CG5948	CG9306	levy	proPO45	ing	Tango7
CG11752	CG30094	CG6055	CG9603	lt	Pros26	CG9034	TfIIA-S-2
CG11885	CG31051	CG6115	CG9782	MED30	Pros29	CG5399	topi
CG12203	CG32112	CG6163	CG9815	MED31,slx1	Prx3	CG18809	tsh
CG12592	CG32165	CG6188	cib	Mgstl	roX2	CG5548	tty
CG13078	CG32650	CG6356	CoVIII	Mlc-c	RpL12	CG1998	TwdlC
CG13349	CG33051	CG6357	CR43785	mRpL12	RpL13	CG11590	twr
CG13559	CG3321	CG6362	cype	mRpL20	RpL18	CG1143	CoVIb
CG13625	CG33228						

Table S2 - D: Genes down-regulated in PARP-1-KD dataset

A2bp1	CG11360	CG30122	CG5776	Dlic	kuz	Pax	Smox
Aats-glupro	CG11418	CG30345	CG5830	dm	kuz	pbl	SMSr
Aats-ile	CG11652	CG3036	CG5955	Dpt	l(1)G0289	Pde11	snoRNA:Or-aca1
Abl	CG11665	CG31033	CG6293	DptB	l(3)05822	PEK	snoRNA:Or-aca3
Acph-1	CG11710	CG3107	CG6330	Drep-1	l(3)76BDr	Pepck	snoRNA:Prp8-a
snoRNA:Psi28S-2876	snoRNA:Psi28S-3327c	snoRNA:Psi28S-2442b	snoRNA:Psi28S-1192b	snoRNA:Psi28S-1135a	snoRNA:Psi18S-1377d	snoRNA:Psi18S-1377b	snoRNA:Psi18S-1347a
Akt1	CG11883	CG31637	CG6509	drongo	lack	pfk	pes
Ald	CG11897	CG3168	CG6707	Drs	lama	PGRP-SB1	l(3)L1231
Ald	CG12004	CG31689	CG6770	dsh	Lamp1	PH4alphaEFB	Dro
Alk	CG12024	CG31729	CG6791	Dyrk3	Lap1	pho	CG6479
alpha-Cat	CG12084	CG31739	CG6966	E2f	Lar	Pi3K21B	snoRNA:Psi28S-1232
alpha-Man-I	CG12173	CG31998	CG7028	eas	lds	Pi4KIIalpha	snoRNA:Psi28S-2179
aop	CG12214	CG32000	CG7139	Eb1	Lectin-28C	Piezo	CG31337
aos	CG12290	CG32037	CG7192	EcR	lgs	Pif1A,Pif1B	Acsl
AP-50	CG12547	CG32043	CG7220	edin	lilli	pigs	CG11737
Apc	CG12567	CG32164	CG7261	eIF4G	LIMK1	pirk	snz
aPKC	CG12576	CG32350	CG7326	eIF4G2	lin19	Pk17E	Spf45
arm	CG1275	CG32549	CG7408	Eip93F	lin-28	Pka-R1	spi
armi	CG13084	CG32758	CG7510	Ela	Liprin-alpha	Pkn	SppL
aru	CG13248	CG32767	CG7573	ens	Lis-1	plexA	sra
ash2	CG13252	CG3301	CG7806	Eph	Lk6	plexB	Sra-1
Atet	CG13283	CG33144	CG7900	Ephrin	lost	Pli	sti
ATP7	CG13384	CG3363	CG8086	Etl1	LpR2	PMCA	stnA,stnB
ATPsyn-beta	CG13458	CG33692 CG43736	CG8195	fax	lqf	Pp2A-29B	su(r)
AttA	CG13603	CG3397	CG8334	fd68A	Lrrk	ppa	Sug
AttB	CG13650	CG34165	CG8405	Fim	lt	PRL-1	Sur
AttB	CG13900	CG34227	CG8475	Flo-2	luna	pst	Swim
AttC	CG14082	CG34349	CG8485	flr	Map205	Pten	sws
AttD	CG14253	CG34383	CG8671	flw	Mbs	Ptp69D	sxc
Axn	CG14291	CG3626	CG8678	foi	Mcr	Pu	Sxl
Bap170	CG1440	CG3651	CG8783	foxo	Mctp	pug	syd
baz	CG14435	CG3678	CG8830	Fps85D	m-cup	put	Syx1A
bbx	CG14446	CG3714	CG9008	Fur1	me31B	Pvr	tai
bel	CG14476	CG3797	CG9086	fws	MED26	Rab10	tamo
beta-Spec	CG14478	CG3961	CG9135	fz2	mei-P26	Rab26	Tango13
bol	CG14567	CG40178	CG9220	Galpha49B	MESR4	Rab30	TER94
bon	CG14619	CG40191	CG9281	GATAd	Met	Rac2	th
bor	CG14621	CG4022	CG9328	Gclc	MFS10	Rapgap1	TH1
brat	CG14695	CG40472	CG9646	gfzf	Miro	raptor	Tm1
bur	CG14741	CG4049	CG9717	Gie	Mkp3	RASSF8	tmod
c11.1	CG15046	CG4080	CG9727	Gint3	Mlc-c	Rbcn-3A	Tob
C1GalTA	CG1513	CG41099	CG9733	gish	Mlf	ref(2)P	Tom70
CalpA	CG15385	CG42240	CG9866	Gk	mmy	Rgl	trbd
Cals	CG15784	CG42354	CG9917	gkt	mnd	rgn	Trn
CaMKII	CG1582	CG42360	CG9935	GNBP2	Mnn1	RhoGAP19D	Tsf1
caps	CG1646	CG42389	CG9992	gol	Mnt	Ric	Tsp26A
CASK	CG1657	CG4259	CHKov2	Graf	mon2	rin	Tsp42Eg
Cbl	CG16700	CG42596	Cip4	GstE6	moody	rl	tud

Cct1	CG16857	CG42637	cnk	Gug	mRpS5	rok	Ubc-E2H
Cct5	CG16868	CG42663	CoRest	hang	msi	Rop	Ubpy
CDase	CG17018	CG42671	coro	Hcf	mspo	roq	Ubqn
cdc14	CG1718	CG42674	Cortactin	Hex-A	mthl1	RpA-70	uex
Cdc42	CG17494	CG42708	cos	Hip14	Mtk	Rpn6	Ugt35a
Cdep	CG17528	CG42715	Cpr	hipk	mtm	Rtf1	Ugt86Da
cdi	CG17544	CG42740	CR44031	how	Mtpalpha	rtGEF	Ugt86Dd
CecA1	CG17549	CG42788	Nxf3	HP5	mub	Rtnl1	Ugt86Di
CecA2	CG17683	CG42807	crp	Hr39	Mvl	Sam-S	Ulp1
CecB	CG17715	CG42821	Crtc	Hsc70-4	myo	Sbf	Unc-115a
ced-6	CG17754	CG43175	Csat	Hsc70-4	MYPT-75D	SCAR	unc-13
Cen	CG17829	CG43236	cta	Hsp23	Nc	scny	Unc-76
Cerk	CG17839	CG43427	Cul-2	Hsp26	nct	scramb1	unk
cert	CG18213	CG43462	CycA	Hsp27	ND75	scrib	Upf1
CG10311	CG18473	CG43658	CYLD	Hsp60	Nipped-B	scyl	Vang
CG10359	CG18547	CG43968	Cyp28d1	Hsp70Bb	nonC	Sdc	Vha55
CG10413	CG18596	CG44006	Cyp6a14	Hsp70Bc	Nsf2	sdk	Vha68-2
CG10426	CG18812	CG4752	Cyp6a17	hyd	NtR	Sec16	vtd
CG10462	CG1882	CG4893	Cyp6a20	Hydr2	nuf	sec71	wapl
CG10492	CG1910	CG4911	Cyp6a21	Iap2	ssp2	sfl	WASp
CG10559	CG1951	CG5009	Cyp6a22	ifc	Oat	Shc	waw
CG10600	CG2051	CG5026	Cyp6a23	IntS3	Obp99a	shg	wech
CG10602	CG2061	CG5059	Cyp6a8	Itp-r83A	Oda	sina	wkd
CG10663	CG2162	CG5098	Cyp6a9	Jheh3	ome	sip2	Wnk
CG10702	CG2177	CG5168	Cyp6d2	kcc	oys	skap	X11L
CG1090	CG2201	CG5235	Cyp6d4	kel	p120ctn	sl	XNP
CG10915	CG2316	CG5261	Cyp9f2	Khc-73	Pak3	sle	yin
CG10960	CG2316	CG5315	dally	kibra	papi	slpr	ZAP3
CG10979	CG2519	CG5521	Dbp80	Klp98A	par-6	Smc5	zfh1
CG11155	CG2918	CG5535	Dgp-1	KrT95D	Parp	smg	zfh2
CG11255	CG2991	CG5613	dikar	krz	path	Smg5	Zip3
CG11357	CG30015	CG5674	disp	kto	Patr-1	smid	CR31144

Table S2-E: Biological processes affected after PARP1-KD

Upregulated GO Biological Process after PARP-1-KD

GO:0006119~oxidative phosphorylation

GO:0042775~mitochondrial ATP synthesis coupled electron transport

GO:0022900~electron transport chain

GO:0042773~ATP synthesis coupled electron transport

GO:0006091~generation of precursor metabolites and energy

GO:0022904~respiratory electron transport chain

GO:0000022~mitotic spindle elongation

GO:0007052~mitotic spindle organization

GO:0051231~spindle elongation

GO:0006412~translation

GO:0007051~spindle organization

Down-regulated GO Biological Process after PARP-1-KD

GO:0006928~cell motion

GO:0000902~cell morphogenesis

GO:0007409~axonogenesis
GO:0060284~regulation of cell development
GO:0048812~neuron projection morphogenesis
GO:0031175~neuron projection development
GO:0048667~cell morphogenesis involved in neuron differentiation
GO:0019731~antibacterial humoral response
GO:0048666~neuron development
GO:0032989~cellular component morphogenesis
GO:0030030~cell projection organization
GO:0030182~neuron differentiation
GO:0000904~cell morphogenesis involved in differentiation
GO:0008360~regulation of cell shape
GO:0019730~antimicrobial humoral response
GO:0048858~cell projection morphogenesis
GO:0007411~axon guidance
GO:0022604~regulation of cell morphogenesis
GO:0032990~cell part morphogenesis
GO:0009617~response to bacterium
GO:0006468~protein amino acid phosphorylation
GO:0051674~localization of cell
GO:0048729~tissue morphogenesis
GO:0006959~humoral immune response
GO:0060429~epithelium development
GO:0002009~morphogenesis of an epithelium
GO:0007242~intracellular signaling cascade
GO:0042742~defense response to bacterium
GO:0051960~regulation of nervous system development

Table S2-F: Biological processes affected after PARylation inhibition

Up-regulated GO Biological Process after PARylation Inhibition

GO:0006351~transcription, DNA-dependent
GO:0032774~RNA biosynthetic process
GO:0006366~transcription from RNA polymerase II promoter
GO:0006412~translation
GO:0006612~protein targeting to membrane

Down-regulated GO Biological Process after PARylation Inhibition

GO:0006959~humoral immune response
GO:0019730~antimicrobial humoral response
GO:0006952~defense response
GO:0006955~immune response
