# Supplementary material for: Involvement of PARP1 in the regulation of alternative splicing
Source: Cell Discov. 2016 Feb 16;2:15046–. doi: 10.1038/celldisc.2015.46 (PMC4860959; doi:10.1038/celldisc.2015.46)
Supplement: Supplementary Table S3 [file celldisc201546-s11.pdf]

Table 3: Genes with ASEs modulated by PARP-1 depletion/ PARylation inhibition as detected by MISO

B52	Pak	mfas	RecQ5	CG7546	CG11266
CG6454	CG11148	Rbp1	RpL10Ab	Trl	CG17721
ZnT63C	iPLA2-VIA	Best1	Irk3	TBPH	vlc
Stat92E	MFS17	CG11357	PMCA	Galpha49B	CG32638
LM408	CG6767	CG17698	spz	Dscam	CG3209
Tep2	ewg	ps	mim	CG17018	eIF-4B
Mef2	Naam	RpS5a	MRP	pst	Saf-B
eRF1	lark	gammaCop	CG3376	Sin	cbt
pUf68	CG32626	CG12567	CG1513	Taf12	CG2201
grass	crq	Hel25E	CG2698	CG32582	CG1161
Ect4	Traf-like	Myo31DF	TFAM	wah	CG7206
CG4822	Nmda1	CG11455	CG4896	betaggt-I	CG3887
pck	Amun	NFAT	crc	trol	Crk
CG43674	CG40178	pbl	CG13900	CAP	Rm62
Cals	RhoGAP71E	CG8176	grp	ctrip	GlcAT-P
mbc	CG30122	CG8671	Ald	Ppn	Parp
Pp4-19C	CG12582	CG17490	CG13124	CG17002	CG8080
mub	su(w[a])	RpL15	MP1	par-1	Uhg3
Bsg	lqf	crol	Sap47	Mvl	Cctgamma
CG40191	CG9705	by	CG10462	CG34133	kis
CG8944	Ranbp16	CG8369	exba	fwe	HmgD
CTPsyn	mrj	Snx6	CG7956	Piezo	CG18659
mod(mdg4)	Pten	l(2)gl	Gel	sip2	CycG
CG3566	Gbeta13F	CG11377	CG42788	CG13760	WRNexo
tacc	CG2371	CG3812	CG42533	CG5059	CG9331
Ehbp1	CG8974	CG7414	CG11791	Rbp2	CoRest
NKAIN	CG8839	CG16952	Fur2	CG40160	CG31955
brat	CG5973	CG5021	CG6982	Wbp2	Pfk
ens	Hsromega	CG5537	CG15093	pallidin	RpS21
CG17734	Akap200	CR43723	CG8786	CG17715	ovo
CR43785	Gr43a	CG8475	CG5515	Atg1	JTBR
Tango14	CG3744	CG7971	dom	Atg18	CG11883
CG15445	CG1516	Tpc1	Moe	achi	tmod
Pep	CaMKII	spi	CG42674	Hk	CG41378
CaMKI	Pif1A	Su(z)12	Ac3	CG6084	Arf51F
Taf1	Mbs	MESR6	mys	vtd	CG32138
CG42708	CG40045	klar	Patronin	CG31638	CG15211
CG32521	Ars2	mth	CG7927	akirin	mud
CG31033	CG32016	CG3689	CG41099	CG1129	PHGPx
eIF-5A	skpA	lawc	Hsc70-3	SP1173	Ire1
CG11686	CG9766	CG3107	CkIIbeta	Pi3K92E	chic
step	Pvr	Haspin	PGRP-LC	CG42596	MESK2
CG43340	PNUTS	Tango2	RpS14a	drpr	Pdp1
CG32576	RpS26	SC35	CG32276	CG17119	Tif-IA
CG9536	CG6805	Eph	CR43496	CG30323	gish
Csp	CG8372	Eip71CD	CG31712	CG13384	MED31
CG3271	CG12213	E2f	CG3065	vig2	rin
qm	Rb97D	mri	Aats-lys	CG33096	CG12163
BubR1	CG42336	eIF-4E	Pp2A-29B	CG8678	Fps85D
eIF4G	CG10353	Lasp	CG11163	CG43954	baz
Rala	CG32068	CG5708	Sin3A	Sam-S	Cdc37
sqd	CG5830	bbc	CG3884	Spn	bl
Rad23	CG6340	zip	Flo-2	E(bx)	Gug
vnc	Hrb98DE	Nhe3	Zn72D	Ctr1A	CG9646
CG17082	CG18769	Atpalpha	CG31550	CG11737	gem
Stlk	chif	tomosyn	mtacp1	spen	pigeon
Tab2	CG13004	CG7139	plexA	Zasp52	rump
rap	CG42678	Slip1	eater	RhoGAPp190	Asator
bmm	CG17471	dre4	Caps	cg	l(1)G0156
itp					

Table 3B: Common genes whose ASEs were detected by MATS and MISO and also was shown in ChIP-seq data to be bound by PARP1 at the internal exon-intron boundary.

CG5746	CG5746 gene product from transcript CG5746-RG	3R
CycG	Cyclin G	3R
KLHL18	CG3571 gene product from transcript CG3571-RC	3R
Trl	Trithorax-like	3L
iPLA2-VIA	calcium-independent phospholipase A2 VIA	3L
CG10249	CG10249 gene product from transcript CG10249-RG	2R
dco	discs overgrown	3R
Hr78	Hormone-receptor-like in 78	3L
CG6923	CG6923 gene product from transcript CG6923-RA	3R
CG7987	CG7987 gene product from transcript CG7987-RA	3R
fl(2)d	female lethal d	2R
DnaJ-H	DnaJ homolog	2L
fdl	fused lobes	2R
Vha16-1	Vacuolar H[+] ATPase 16kD subunit 1	2R
CG42708	CG42708 gene product from transcript CG42708-RA	2R
RpS13	Ribosomal protein S13	2L
barr	barren	2L
CG6767	CG6767 gene product from transcript CG6767-RA	3L
eIF-4E	Eukaryotic initiation factor 4E	3L
exu	exuperantia	2R
CG10151	CG10151 gene product from transcript CG10151-RA	2R
SelR	CG6584 gene product from transcript CG6584-RI	3R
Prm	Paramyosin	3L
achi	achintya	2R
