# Supplementary material for: Involvement of PARP1 in the regulation of alternative splicing
Source: Cell Discov. 2016 Feb 16;2:15046–. doi: 10.1038/celldisc.2015.46 (PMC4860959; doi:10.1038/celldisc.2015.46)
Supplement: Supplementary Table S4 [file celldisc201546-s12.pdf]

**Table S4: Common genes whose ASEs were detected by MATS and also was shown in ChIP-seq data to be bound by PARP1 at the internal exon-intron boundary.**

## 1 Peak Calling using MACS

In the second step, we used MACS (Model-based Analysis for ChIP-Seq) to identify peaks from the ChIP-Seq data and therefore determine PARP1 binding sites. For each replicate (and combined), we identified peaks using five p-value thresholds: 1e-5, 1e-4, 1e-3, 1e-2, and 0.05. The table below shows the number of identified peaks for each replicate and combined replicates.

**A: Number of peaks identified for each replicate and combined replicates.**

| Sample | 1e-5 | 1e-4 | 1e-3 | 1e-2 | 0.05 |
|---|---|---|---|---|---|
| PARP1 Rep. 1 | 3723 | 4996 | 6988 | 9944 | 12820 |
| PARP1 Rep. 2 | 1647 | 2385 | 4029 | 8820 | 16406 |
| PARP1 Rep. 3 | 4258 | 5589 | 7420 | 10208 | 12493 |
| PARP1 Rep. 4 | 2296 | 2794 | 3686 | 5673 | 7903 |
| Combined | 3273 | 4122 | 5350 | 7056 | 8590 |

## 2 Overlap Peaks with Significant AS Events

To determine whether there were PARP-1 binding sites associated with the significant events generated by MATS, we computed the number of peaks (per replicate and combined) that correspond to each event for each comparison. The following tables show for each comparison the number of peaks that overlap with each event using combined PARP1 replicates.

*Significant events found with MATS Combined:*

**B: Number of events covered by one or more peaks for Control_vs_PARP1KD**

| SE | MXE | A5SS | A3SS | RI |
|---|---|---|---|---|
| 122 | 75 | 31 | 46 | 88 |

| p-value | SE | MXE | A5SS | A3SS | RI |
|---|---|---|---|---|---|
| 1e-5 | 13 | 11 | 1 | 3 | 11 |
| 1e-4 | 16 | 13 | 1 | 4 | 17 |
| 1e-3 | 22 | 20 | 1 | 10 | 24 |
| 1e-2 | 39 | 32 | 5 | 14 | 33 |
| 0.05 | 43 | 41 | 8 | 16 | 34 |

**C: Number of events covered by one or more peaks for Control_vs_PARylation**

| SE | MXE | A5SS | A3SS | RI |
|---|---|---|---|---|
| 173 | 55 | 54 | 38 | 100 |

| p-value | SE | MXE | A5SS | A3SS | RI |
|---|---|---|---|---|---|
| 1e-5 | 20 | 14 | 2 | 2 | 8 |
| 1e-4 | 26 | 15 | 4 | 4 | 16 |
| 1e-3 | 34 | 20 | 5 | 8 | 20 |
| 1e-2 | 48 | 22 | 11 | 12 | 25 |
| 0.05 | 60 | 25 | 14 | 12 | 29 |

**D: Number of peaks overlapping with all genes**

| Total Genes | 1e-5 | 1e-4 | 1e-3 | 1e-2 | 0.05 |
|---|---|---|---|---|---|
| 15682 | 2211 | 3051 | 4331 | 6062 | 7576 |
