# Supplementary material for: Involvement of PARP1 in the regulation of alternative splicing
Source: Cell Discov. 2016 Feb 16;2:15046–. doi: 10.1038/celldisc.2015.46 (PMC4860959; doi:10.1038/celldisc.2015.46)
Supplement: Supplementary Table S5 [file celldisc201546-s13.pdf]

Table S5: proteins detected by Mass spectrometry after PAR-CLiP experiments. Columns 3-5 the proteins detected in the various experimental conditions: (normal PAR-CliP, stringent RNase A and stringent DNase1 digestion respectively) and the amounts). *(+) Indicates presence in the experimental condition while (-) represents absence. Significant increase in the various conditions compared to normal PAR-CLiP experimental condition), is depicted by the number of ++++. And significant decrease compared to normal PAR-CLIP experimental condition is depicted by the number of - - -. Column 5 (Function) shows known functions of the protein, Columns 6 and 7, shows experimentally confirmed evidence whether this protein is a PARP1 interactor or found in the spliceosomal complex respectively.*

	Protein name	Normal	Stringent RNase	Stringent DNase	Known Function and references	PARP1 interactor	spliceosomal complex
**RNA metabolism**							
Q9NR30	Nucleolar RNA helicase (DDX21)	++	-	-		✓	✓
O15523	ATP-dependent RNA helicase	++	-	+++		✓	✓
P19338	Nucleolin	+	-	-	Colocalizes with splicing factors [3], [4]	✓	
P0C0L4	Complement C4-A	+++	++	+			
P0C0L5	Complement C4-B	+++	++	+			
P43243	Matrin-3	+	-	-	Stabilizes mRNA [5]		✓
Q6PKG0	La-related protein	+++	++	-			✓
P57081	tRNA(guanine-N(7))-methyltransferase subunit	+	-	+	tRNA capping		
Q15139	Serine/threonine-protein kinase D1	+	-	+	Modulates subnuclear distribution of pre-mRNA splicing factors [6]	✓	✓
Q94806	Serine/threonine-protein kinase D3	+	-	+	Modulates subnuclear distribution of pre-mRNA splicing factors [6]	✓	✓
P62847	40S ribosomal protein S24	+	+	-			
P62753	40S ribosomal protein S6	++	-	++			
P23396	40S ribosomal protein S3	+	+	-		✓	✓
P62241	40S ribosomal protein S8	+	-	++		✓	✓
P46781	40S ribosomal protein S9	+	++	+		✓	✓
P61313	60S ribosomal protein L15	+	-	-			
Q02878	60S ribosomal protein L6	+	-	+			
P50914	60S ribosomal protein L14	++	+	++		✓	
P26373	60S ribosomal protein L13	++	+++	++++			
P84098	60S ribosomal protein L19	++	++	++			
P47914	60S ribosomal protein L29	++	-	-			
P42766	60S ribosomal protein L35	+	+	+			
P46779	60S ribosomal protein L28	+	-	+		✓	
**DNA replication and Transcription regulation**							
P19338	Nucleolin	+	-	-	Histone chaperone [7]		✓
P06748	Nucleophosmin	+	+++	++	[8,9]		✓
O15523	ATP-dependent RNA helicase	++	-	+++	Interacts with Pol II [10-12]; DNA topoisomerase [13]		

P13639	Elongation factor 2	+	-	-			
P62805	Histone 4	+	++	+	[14]	✓	
P16104	Histone H2Ax	+	-	-			
P0C0S5	Histone H2Az	+	-	-			
Q14997	Proteasome activator complex subunit 4	+	+	-	Acetylation-mediated degradation of histone [15]		
Q03701	CCAT/enhancer-binding protein zeta	+	-	-	Transcription activation[16,17]		✓
Q9Y566	SH3 and multiple ankyrin repeat domain protein 1	+	+	-			
Q9H174	WD repeat-containing protein 13	+	+	-	snRNPs aids in the association of U5 and U6 snRNPs [18]		
Q5T4T6	Synaptonemal-complex protein 2	+	+	-			
Q9HCE0	UPF0493 protein KIAA1632	+	+	-			
Q776K3	Protein prenyltransferase alpha subunit	+	+	-			
**Cell cycle**							
Q15042	Rab3 GTPase-activating protein catalytic subunit	+	-	-	Binds to chromosomes [19]	✓	
**Cell death**							
P63104	14-3-3 protein zeta	++	++	-	Recognizes a histone code [20] [21]	✓	
**Others**							
P04196	Histidine-rich glycoprotein	++	+	+			
P55072	Transitional endoplasmic reticulum ATPase	+	-	-			
Q8N3F9	Integral membrane protein GPR137C	+	+	-			
Q14746	Conserved oligomeric Golgi complex subunit	+	-	-			
Q06033	Inter-alpha-trypsin inhibitor heavy chain H3	+	+	++			

1. Isabelle, M. *et al.* Investigation of PARP-1, PARP-2, and PARG interactomes by affinity-purification mass spectrometry. *Proteome Sci* **8**, 22 (2010).
2. Rappsilber, J., Ryder, U., Lamond, A.I. & Mann, M. Large-scale proteomic analysis of the human spliceosome. *Genome Res* **12**, 1231-45 (2002).
3. Das, S. *et al.* Characterization of nucleolin K88 acetylation defines a new pool of nucleolin colocalizing with pre-mRNA splicing factors. *FEBS Lett* **587**, 417-24 (2013).
4. Mi, Y. *et al.* Apoptosis in leukemia cells is accompanied by alterations in the levels and localization of nucleolin. *J Biol Chem* **278**, 8572-9 (2003).
5. Salton, M. *et al.* Matrin 3 binds and stabilizes mRNA. *PLoS One* **6**, e23882 (2011).
6. Misteli, T. & Spector, D.L. Serine/threonine phosphatase 1 modulates the subnuclear distribution of pre-mRNA splicing factors. *Mol Biol Cell* **7**, 1559-72 (1996).
7. Angelov, D. *et al.* Nucleolin is a histone chaperone with FACT-like activity and assists remodeling of nucleosomes. *EMBO J* **25**, 1669-79 (2006).
8. Meder, V.S., Boeglin, M., de Murcia, G. & Schreiber, V. PARP-1 and PARP-2 interact with nucleophosmin/B23 and accumulate in transcriptionally active nucleoli. *J Cell Sci* **118**, 211-22 (2005).
9. Borggrefe, T., Wabl, M., Akhmedov, A.T. & Jessberger, R. A B-cell-specific DNA recombination complex. *J Biol Chem* **273**, 17025-35 (1998).
10. Pellizzoni, L., Charroux, B., Rappsilber, J., Mann, M. & Dreyfuss, G. A functional interaction between the survival motor neuron complex and RNA polymerase II. *J Cell Biol* **152**, 75-85 (2001).

11. Anderson, S.F., Schlegel, B.P., Nakajima, T., Wolpin, E.S. & Parvin, J.D. BRCA1 protein is linked to the RNA polymerase II holoenzyme complex via RNA helicase A. *Nat Genet* **19**, 254-6 (1998).
12. Nakajima, T. *et al.* RNA helicase A mediates association of CBP with RNA polymerase II. *Cell* **90**, 1107-12 (1997).
13. Zhou, K. *et al.* RNA helicase A interacts with dsDNA and topoisomerase IIalpha. *Nucleic Acids Res* **31**, 2253-60 (2003).
14. D'Amours, D., Desnoyers, S., D'Silva, I. & Poirier, G.G. Poly(ADP-ribosyl)ation reactions in the regulation of nuclear functions. *Biochem J* **342 (Pt 2)**, 249-68 (1999).
15. Qian, M.X. *et al.* Acetylation-mediated proteasomal degradation of core histones during DNA repair and spermatogenesis. *Cell* **153**, 1012-24 (2013).
16. Lum, L.S., Sultzman, L.A., Kaufman, R.J., Linzer, D.I. & Wu, B.J. A cloned human CCAAT-box-binding factor stimulates transcription from the human hsp70 promoter. *Mol Cell Biol* **10**, 6709-17 (1990).
17. Choudhary, C. *et al.* Lysine acetylation targets protein complexes and co-regulates major cellular functions. *Science* **325**, 834-40 (2009).
18. Chan, S.P., Kao, D.I., Tsai, W.Y. & Cheng, S.C. The Prp19p-associated complex in spliceosome activation. *Science* **302**, 279-82 (2003).
19. Capalbo, L., D'Avino, P.P., Archambault, V. & Glover, D.M. Rab5 GTPase controls chromosome alignment through Lamin disassembly and relocation of the NuMA-like protein Mud to the poles during mitosis. *Proc Natl Acad Sci U S A* **108**, 17343-8 (2011).
20. Winter, S. *et al.* 14-3-3 proteins recognize a histone code at histone H3 and are required for transcriptional activation. *EMBO J* **27**, 88-99 (2008).
21. Healy, S., Khan, D.H. & Davie, J.R. Gene Expression Regulation Through 14-3-3 Interactions with Histones and HDACs. *Discovery Medicine* **59**, 349-358 (2011).
