# Supplementary material for: Involvement of PARP1 in the regulation of alternative splicing
Source: Cell Discov. 2016 Feb 16;2:15046–. doi: 10.1038/celldisc.2015.46 (PMC4860959; doi:10.1038/celldisc.2015.46)
Supplement: Supplementary Table S6 [file celldisc201546-s14.pdf]

Table S6: Primers used in this study

Primers for semi-quantitative PCR to measure alternative spliced isoforms

CAPT-1-Alternate exon<br>For: 5'-GCCATTTGGTTCTGTGCTTGGTCG-3'	 Rev:5'-AGGCGCTCTTCACGAGCTCC-3'
CAPT-1-Constitutive exon<br>For: 5'-CAACTGCAGTACGTGACGCT-3'	 Rev:5'-GTCGCCAGTGACCTTCTTCA-3'
ACHI-Alternative exon<br>For: 5'-TGTTGTTTGGCACGCAGCAAGG-3'	 Rev:5'-TCCGGCGAGATCATTGCAGCTC-3'
ACHI-constitutive exon<br>For: 5'-AAGGCGAAGAGAGTGCACAG-3'	 Rev: 5'-GGCACGCTCATCTGGACTTG-3'
DNAJH1-alternative exon<br>For: 5'-AGCCCCTTCGATACG-3'	 Rev: 5'-TAGCGTGGCTAATGTATTAGT-3'
DNAJH1-constitutive exon<br>For: 5'-AACTACCGAAAATTGGCAAA-3'	 Rev: 5'TAGCGTGGCTAATGTATTAGT-3'
G32196-alternative exon<br>For: 5'-AACTTGCCGAGCAGTGTAGC-3'	 Rev: 5'-CCCAAATGGCTCCGTACACA-3'
CG32196-constitutive exon<br>F: 5'-CGAAAAATTGGCTAGGCGCTC-3'	 Rev: 5'-GCACTTGTTTGCCATTGTGC-3'
CG2915-alternative exon<br>For: 5'-GCGTTCTCTATAATCCAGCCCT-3'	 Rev: 5'-TTGCGGTCGATCTTCTCCTG-3'
DGO-alternative exon<br>For: 5'-TCTGGGCGTTCCATCAATCC-3'	 Rev: 5'-CCTCATGGAGCGGGGTATTC-3'
DGO-constitutive exon<br>For: 5'-ATGGAGATACTCCGCTGCAC-3'	 Rev: 5'-CGGAAGAAGGGACCGCAATA-3'
BARR-alternative exon<br>For:5'-AAATTGCGGCGTCGTTTGAA-3'	 Rev: 5'-CGCATGGCGTCCAAGTAGAT-3'
BARR-constitutive exon<br>For:5'-GCGTGTGGACTCCATCTACT-3'	 Rev: 5'-TGATGTAGCCCGTATGCAGC-3'
CG10625-alternative exon<br>For: 5'-CGACCAAAAGACAACGAACCG-3'	 Rev: 5'-AACGTGGACATATGGGGGTG-3'
Primers for quantitative RT-PCR to measure isoforms	
PARP-1<br>For: 5'–TCGACGTGTCGTGGATGTGAACAA3';	 Rev: 5' ACAAAGGTTGGCCTCCGTACTTCA-3'
β-ACTIN<br>For: 5'–TCGCGAATTTGACCGACTACCTGAT-3';	 Rev: 5'-TTGATGTCACGGACGATTTCCGC-3'
ACHI-1<br>For: 5'–TGGCCTCGATACTACGGTTG3';	 Rev: 5'–GGTTTTCCCAGTGTTTCAGC-3'
ACHI-2<br>For: 5'–AACTCCAGTCCTGCCACATC3';	 Rev: 5'–TCTTCGTCATCGAACTCTGTCTGG-3'
CAPT-1<br>For: 5'–GTGTTCGCTACGCAGCTTCT-3';	 Rev: 5'–CTGCTGATGAATTTCGAAGG-3'
CAPT-2<br>For: 5'–ACACGCCTGCAAAATGTTCT-3';	 Rev: 5'–CCGCTTTTCCTTCTCCTTCT-3'
STAU-1<br>For: 5'–CCATCGATCGAACGGAGTAT-3' ;	 Rev: 5'– TTTTCGTACGCAATGGTCAC-3'
FL2D-1<br>For: 5'–TACGCGAAATCAACGTTTG-3';	 Rev: 5'–CTCCAATACCACACGCACAC-3'
Fl2D-2<br>For: 5'–TGCAAATAATGGCAGGTGTT-3';	 Rev: 5'–TCGATTTCGATTGCTTCGTT-3'
DNAJ1-1<br>For: 5'–CGTTAGCACTTTCCACACACGA-3';	 Rev: 5'–CAAGATGGCACACGTAGCTG-3'
DNAJ1-2<br>For: 5'–CTTGTTAGCCAATATGACTTGAAAA-3';	 Rev: 5'-ACGTCGTATAAATTTAGGTTGTCCA-3'

Drosophila siRNA used for KD2: DRSC07725: dsRNA position relative to Exon 4: 2 – 274
CCAATAAAATCTGAAGAACTACCAGATACAAAAAGAGCTAAGATGGAATTATCAGATACAAAT
GAAGAAGGAGAAAAGAAACAACGCTTAAAAGATCAAAATGATGCCTACTTCAGGTTTCGCGA
TGACATTAAAAATAAAATGAAGAAGAAAGACATTGATATACTTCTAAAGTTTAATAATCAACAA
CCTGTAACTGGTGACACAGAAAAGTTATTTGATCAAACTGCCGATTTACTGACATTCGGAGCT
ATTGAATCATGTTCTGAATGC

Human siRNA used for PARP1 knockdown was from Dharmacon. To deplete endogenous PARP1, we used siRNAs targeting different regions in PARP1 (si-PARP1), which are designed to minimize the off-target effects (ON-TARGETplus, Thermo Scientific Dharmacon).

Primers used for qRT-PCR to measure differential gene expression patterns	
CAM	
For: 5’-GCACAGTTATGCGCTCCCT-3	Rev: 5’-GCGTGCCATCATGGTAAGGAA-3’
OAT	
For: 5’-GCGTGTTTGTCTGGGATGTG-3’	Rev: 5’-CTACGATCTTGGGATGGCAGT-3’
WGN	
For: 5’-GGAGGAGCCATGCGTAGTC-3’	Rev: 5’-GCAGAGCCAGGTAACTGCC-3’
HMGZ	
For: 5’-GTCCCCTTTCCGCCTACATG-3’	Rev: 5’-CTTGATCTGTTCGCGGGTCT-3’
LOST	
For: 5’-CAGCCCATAGAGCCCACAAAG-3’	Rev: 5’-AATGCCAACCTTGCCCTCTT-3’
E2F1	
For: 5’-TACGATCACGTCCTGAGCAG-3’	Rev: 5’-CCCTTGTCCAGACTGATGTAGA-3’
ASF1	
For: 5’-CCATTGTTCTGCTTACCTGCT-3’	Rev: 5’-GGTCAGCGTAGTCGTTATTCAC-3’
PEX11	
For: 5’-AAGCAAGCTGTCACAATCGCT-3’	Rev: 5’-TAGAAGTCCCGGCACAAGTTC-3’
SLOW	
For: 5’-CCTGCTGCTCTGTTGCACA-3’	Rev: 5’-GGATGAGGGCTGGTGCTATTG-3’
CECA	
For: 5’-AAGCTGGGTGGCTGAAGAAA-3’	Rev: 5’-TGTTGAGCGATTCCCAGTCC-3’
